# Phosphorylation of the receptor protein Pex5p modulates import of proteins into peroxisomes

**DOI:** 10.1515/hsz-2022-0168

**Published:** 2023-02-23

**Authors:** Sven Fischer, Jérôme Bürgi, Shiran Gabay-Maskit, Renate Maier, Thomas Mastalski, Eden Yifrach, Agnieszka Obarska-Kosinska, Markus Rudowitz, Ralf Erdmann, Harald W. Platta, Matthias Wilmanns, Maya Schuldiner, Einat Zalckvar, Silke Oeljeklaus, Friedel Drepper, Bettina Warscheid

**Affiliations:** Biochemistry and Functional Proteomics, Institute of Biology II, Faculty of Biology, University of Freiburg, Schänzlestrasse 1, D-79104 Freiburg, Germany; Hamburg Unit c/o DESY, European Molecular Biology Laboratory (EMBL), Notkestrasse 85, D-22607 Hamburg, Germany; University Medical Center Hamburg-Eppendorf, Martinistrasse 52, D-20246 Hamburg, Germany; Department of Molecular Genetics, Weizmann Institute of Science, Rehovot 7610001, Israel; Biochemistry of Intracellular Transport, Medical Faculty, Ruhr University Bochum, D-44780 Bochum, Germany; Systems Biochemistry, Medical Faculty, Ruhr-University Bochum, D-44780 Bochum, Germany; Biochemistry II, Theodor Boveri-Institute, Biocenter, University of Würzburg, Am Hubland, D-97074, Würzburg, Germany; Signalling Research Centres BIOSS and CIBSS, University of Freiburg, D-79104 Freiburg, Germany

**Keywords:** high-content screen, mass spectrometry, Pex5p TPR domain, posttranslational modification, protein localization

## Abstract

Peroxisomes are organelles with vital functions in metabolism and their dysfunction is associated with human diseases. To fulfill their multiple roles, peroxisomes import nuclear-encoded matrix proteins, most carrying a peroxisomal targeting signal (PTS) 1. The receptor Pex5p recruits PTS1-proteins for import into peroxisomes; whether and how this process is posttranslationally regulated is unknown. Here, we identify 22 phosphorylation sites of Pex5p. Yeast cells expressing phospho-mimicking Pex5p-S507/523D (Pex5p^2D^) show decreased import of GFP with a PTS1. We show that the binding affinity between a PTS1-protein and Pex5p^2D^ is reduced. An *in vivo* analysis of the effect of the phospho-mimicking mutant on PTS1-proteins revealed that import of most, but not all, cargos is affected. The physiological effect of the phosphomimetic mutations correlates with the binding affinity of the corresponding extended PTS1-sequences. Thus, we report a novel Pex5p phosphorylation-dependent mechanism for regulating PTS1-protein import into peroxisomes. In a broader view, this suggests that posttranslational modifications can function in fine-tuning the peroxisomal protein composition and, thus, cellular metabolism.

## Introduction

Peroxisomes are dynamic organelles of eukaryotic cells that fulfill a variety of essential metabolic functions. A common feature of peroxisomes is fatty-acid β-oxidation and the degradation of hydrogen peroxide resulting from various oxidative reactions (Wanders and Waterham 2006). Species- and/or tissue-specific functions include the production of penicillin and amino acid metabolism in fungi (Breitling et al. 2002; Kiel et al. 2005b; Sprote et al. 2009) or the generation of plasmalogens and bile acids in humans (Wanders and Waterham 2006). The vital importance of peroxisomes is emphasized by the occurrence of severe, and often lethal, disorders in humans with dysfunctional peroxisomes (Cipolla and Lodhi 2017; Waterham et al. 2016).

Peroxisomal matrix proteins are nucleus-encoded and need to be imported into the organelle. This process generally relies on the recognition of distinct peroxisomal targeting signals (PTS) by specific cytosolic receptor proteins (Walter and Erdmann 2019). The majority of peroxisomal matrix proteins contain a carboxy-terminal PTS1 (the last three amino acids being serine-lysine-leucine (SKL) or variants of it), which is recognized by Pex5p (Gould et al. 1989; van der Leij et al. 1993). Most PTS1-proteins bind to Pex5p via a highly conserved tetratricopeptide repeat (TPR) domain, which is located in the carboxy-terminal region of the receptor and consists of seven consecutive TPR segments (TPR1-TPR7) (Brocard et al. 1994; Stanley et al. 2007). At the peroxisomal membrane, cargo-loaded Pex5p binds to Pex14p of the docking complex (in *Saccharomyces cerevisiae* consisting of Pex13p, Pex14p, and Pex17p; Agne et al. 2003), which is mediated by conserved WxxxF motifs in the amino-terminal region of Pex5p (Otera et al. 2002). Subsequently, Pex5p and Pex14p form a highly dynamic import pore through which the cargo is released into the peroxisomal matrix (Meinecke et al. 2010). Pex5p is then either monoubiquitinated at residue cysteine 6, resulting in receptor recycling (Platta et al. 2014) following swift deubiquitination (El Magraoui et al. 2019), or polyubiquitinated at K18/K24, which marks the receptor for proteasomal degradation (Kiel et al. 2005a; Platta et al. 2004, 2007).

In addition to cargo proteins following the Pex5p-import route, few proteins are imported via Pex7p, which recognizes a different PTS (PTS2; Lazarow 2006), or by the Pex5p homolog Pex9p (Effelsberg et al. 2016; Yifrach et al. 2016). Some proteins can also indirectly be associated to the targeting receptor for import by piggybacking (Effelsberg et al. 2015; Gabay-Maskit et al. 2020; Glover et al. 1994; McNew and Goodman 1994).

Protein import into peroxisomes is a dynamic process: depending on the metabolic conditions of the cells, the localization of proteins may be shifted between the cytosol and peroxisomes, as reported for enzymes of the glyoxylate cycle (Kunze et al. 2002; Schummer et al. 2020). Furthermore, proteins that are targeted to peroxisomes via Pex5p exhibit different targeting priorities that may vary depending on the metabolic condition (Rosenthal et al. 2020). This implies that peroxisomal matrix protein import needs to be regulated. For yeast, it has been shown that phosphorylation of the PTS2 protein Gpd1p promotes its import into peroxisomes (Jung et al. 2010), and peroxisomal import of Cit2p is modulated by phosphorylation of Pex14p (Schummer et al. 2020). Furthermore, in human cells, phosphorylation of PEX14 modulates peroxisomal import of catalase (Okumoto et al. 2020). However, with these exceptions, post-translational mechanisms that allow to directly modulate and fine-tune the import of newly synthesized matrix proteins from the cytosol into peroxisomes are unknown so far.

Here, we uncover a new regulatory layer and show that peroxisomal import of PTS1-proteins mediated by Pex5p can be modulated at the posttranslational level. Using high-resolution mass spectrometry (MS), we identified 22 phosphorylation sites of Pex5p in *S. cerevisiae*. We generated various Pex5p phosphosite mutants and found that cells expressing the phospho-mimicking mutant Pex5p-S507D/523D (Pex5p^2D^) exhibit reduced levels of import of a green fluorescent protein (GFP), carrying a PTS1 at its carboxy-terminus (GFP-SKL). Notably, both phosphorylation sites are located in the TPR domain of Pex5p that is involved in PTS1-cargo binding. Isothermal titration calorimetry (ITC) and native MS analyses of heterodimeric receptor-cargo complexes showed a considerably lower binding affinity between the PTS1-protein Pcs60p and Pex5p^2D^ compared to Pex5p^WT^. Studying the effect of the Pex5p phospho-mimicking mutations on known PTS1-proteins in *S. cerevisiae* revealed that peroxisomal import of a subset of proteins is strikingly affected, whereas others exhibit reduced import, or are not affected at all. Using fluorescence anisotropy, we identify the extended PTS1, comprising a stretch of amino acid residues directly upstream of the conserved tripeptide (Brocard and Hartig 2006; Fodor et al. 2012; Lametschwandtner et al. 1998), as central region displaying reduced binding to phospho-mimicking Pex5p^2D^. Differences in the binding affinity of extended PTS1 sequences to Pex5p correlated to the observed import phenotype of the corresponding PTS1-protein. Thus, we conclude that phosphorylation of Pex5p in its TPR domain modulates cargo recognition and binding of PTS1-proteins, which presumably constitutes a so far unknown posttranslational mechanism for regulating the flow of newly synthesized matrix proteins into peroxisomes.

## Results

### Identification of Pex5p phosphorylation sites

**Figure 1:**
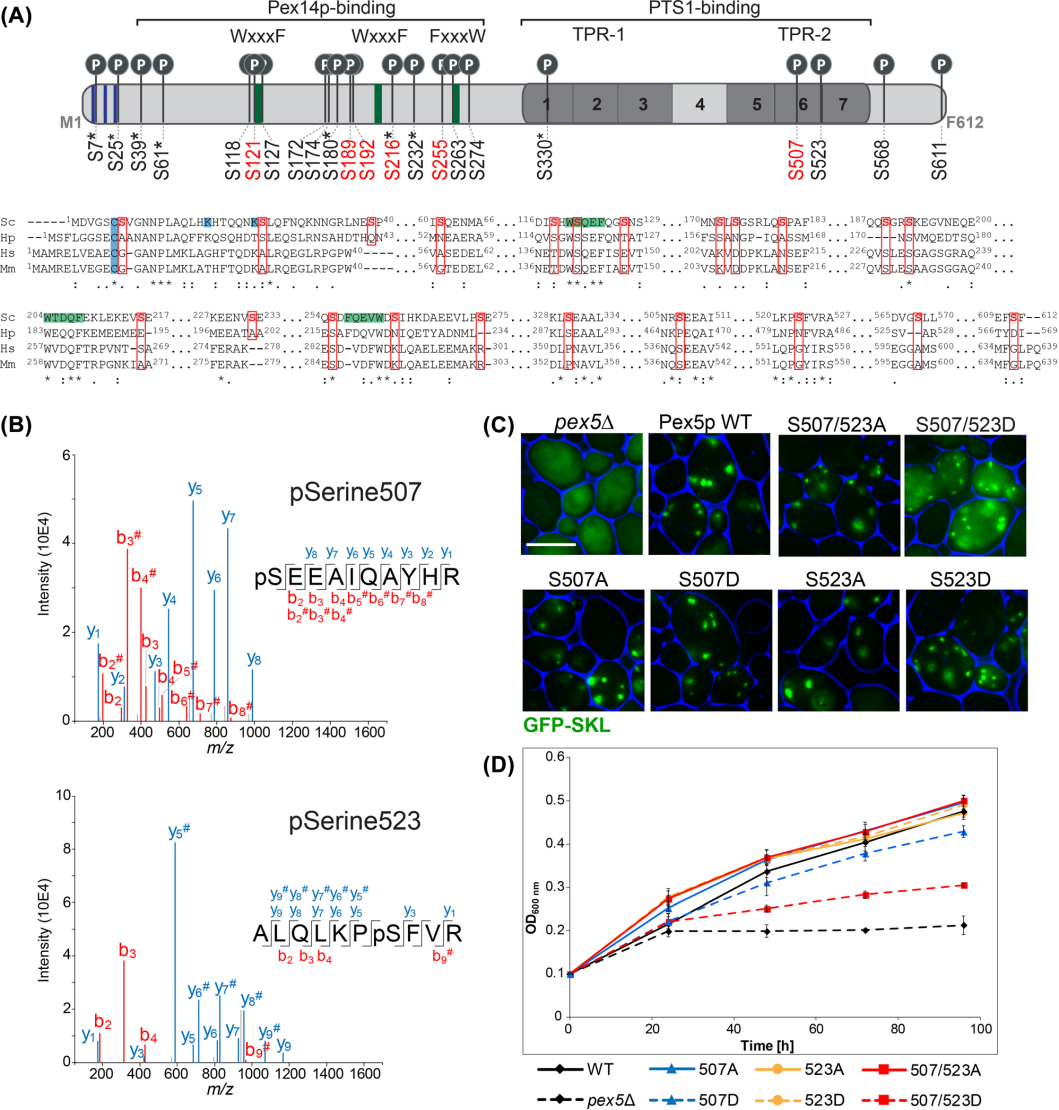
Effects of Pex5p phosphosite mutants on PTS1-protein import and cell growth.(A) Top, Pex5p *in vivo* phosphosite map established by high-resolution mass spectrometry (MS). Red font, overall conserved sites in human; green, conserved WxxxF/FxxxW motifs; blue, mono- and polyubiquitination sites at C6 and K18/K24, respectively; TPR, tetratricopeptide repeat; TPR-1 and TPR-2, first and second TPR triplet; PTS1, peroxisomal targeting signal 1; *known phosphorylation sites. Bottom, Multiple protein sequence alignment of Pex5p from *Saccharomyces cerevisiae* (Sc) with its homologs from *Hansenula polymorpha* (Hp), *Homo sapiens* (Hs) and *Mus musculus* (Mm). Shown are segments of the sequences containing the Pex5p phosphorylation sites for *S. cerevisiae* identified in this work (highlighted in red). (B) Representative MS/MS fragmentation spectra of phosphopeptides with y- and b-ion series as indicated showing phosphorylation of Pex5p at S507 (top) and S523 (bottom). ^#^Fragment ions exhibiting neutral loss of H_3_PO_4_; *m/z*, mass-to-charge ratio. (C) Fluorescence microscopy images of yeast cells grown on oleate and expressing plasmid-encoded Pex5p wildtype or phosphosite mutants (S exchanged to A or D) as indicated and PTS1-tagged GFP (GFP-SKL) in a *PEX5* gene deletion background. Cell boundaries are highlighted in blue. Scale bar, 5 µm. (D) Growth of yeast cells, which were used for fluorescence microscopy experiments shown in 1C, in oleate-containing medium. Time t_0_ marks the point when the cells were shifted from SC to YNO medium and adjusted to an OD_600_ of 0.1. Error bars indicate standard deviation (*n* = 3).

To investigate a potential phosphorylation-dependent regulation of peroxisomal matrix protein import, we mapped phosphorylation sites of the main peroxisomal matrix protein receptor Pex5p in *S. cerevisiae*. We affinity-purified Pex5p from cells expressing Pex5p fused to a carboxy-terminal Tobacco Etch Virus (TEV) protease-cleavable Protein A (TPA) tag from its native chromosomal location. The functionality of TPA-tagged Pex5p was shown in previous reports (Kerssen et al. 2006; Schafer et al. 2004). Phosphopeptides of Pex5p were enriched using titanium dioxide beads (Thingholm et al. 2006) and analyzed by MS to identify and localize individual phosphorylation sites. As a result, we determined 22 unique phosphosites at serine residues in Pex5p, of which 14 are reported here for the first time (Figure 1A, top; Supplementary Table 1). Eight residues were previously identified in global phosphoproteomic studies with unknown function (pS7, pS25, pS39, pS61, pS180, pS216, pS232, pS330) (Albuquerque et al. 2008; Dokladal et al. 2021; Hu et al. 2019; Hollenstein et al. 2021; Schreiber et al. 2012). The correct assignment of each phosphosite was corroborated by manual inspection of fragmentation spectra as shown for the newly identified Pex5p phosphosites at S507 and S523 (Figure 1B). A multiple protein sequence alignment revealed conservation of six serine residues, shown to be phosphorylated in *S. cerevisiae*, in human PEX5 (i.e. positions 121, 189, 192, 216, 255, and 507 in yeast Pex5p; Figure 1A, bottom). Furthermore, the phosphosite S118 in yeast corresponds to a threonine in human PEX5, which is also a phosphorylatable residue (Figure 1A, bottom). The majority of identified phosphosites are positioned within the unstructured amino-terminal region of Pex5p, which is involved in Pex14p-binding (Otera et al. 2002; Williams et al. 2005). We further detected phosphorylation at S7 and S25, which are located in close proximity to the mono- and polyubiquitination sites of Pex5p at C6 (Williams et al. 2007) and K18/24 (Platta et al. 2007), respectively. Moreover, five phosphosites (pS330, pS507, pS523, pS568, pS611) were found in the carboxy-terminal half of Pex5p, of which the first three are located within the highly conserved TPR domain shown to be involved in binding of cargo proteins via their PTS1 (Figure 1A, top) (Stanley et al. 2007).

### Pex5p-S507/523D affects peroxisomal import of GFP-SKL

To examine the impact of distinct Pex5p phosphorylation sites on the import of peroxisomal PTS1-proteins, we generated different Pex5p phosphosite mutants in which ten individual sites, two dual sites (S216/232, S507/523) or two clusters of serine residues (Supplementary Figure 1A) were changed to alanine (A) and aspartate (D) to mimic the non-phosphorylated and phosphorylated forms, respectively. Pex5p wildtype (Pex5p^WT^) and phosphosite mutants, expressed from a plasmid under control of the native *PEX5* promoter, were introduced into *pex5*Δ yeast cells expressing plasmid-borne GFP-SKL, an artificial PTS1 cargo protein. Cells were grown in oleate to induce peroxisome proliferation and the subcellular distribution of GFP-SKL was monitored by fluorescence microscopy. Cells lacking Pex5p are deficient in PTS1-protein targeting and subsequent import. Consequently, *pex5*Δ cells show a diffuse fluorescence staining indicative of a cytosolic localization of GFP-SKL (Figure 1C). Upon reintroduction of Pex5p^WT^ into *pex5*Δ cells, import of GFP-SKL is restored as demonstrated by a punctate staining characteristic of peroxisomes (Figure 1C). All strains expressing Pex5p S-to-A/D mutants showed a Pex5p wildtype-like distribution of GFP-SKL (Figure 1C and Supplementary Figure 1B), except for Pex5p^2D^, the phospho-mimicking S507/523D double site mutant (Figure 1C). In these cells, we observed both a punctate staining and diffuse cytosolic fluorescence, suggesting a reduced import of GFP-SKL into peroxisomes (Figure 1C, S507/523D). This effect was only visible for the Pex5p^2D^ mutant, but not for the phospho-mimicking single site mutants Pex5p^S507D^ and Pex5p^S523D^ (Figure 1C). Immunoblot analysis of cells used for the fluorescence microscopy experiments confirmed similar steady-state expression levels for Pex5p^WT^, the Pex5p^2A^ or Pex5p^2D^ variant, and for GFP-SKL in all three strains (Supplementary Figure 1C), ruling out that the phenotype observed for cells expressing Pex5p^2D^ is based on differences in Pex5p or GFP-SKL expression. In accordance with a reduced peroxisomal matrix protein import, Pex5p^2D^-expressing cells exhibited a significant growth defect when cultivated in oleate as sole carbon source (a condition in which peroxisomes become essential) (Figure 1D), whereas growth on glucose was equivalent to Pex5p^WT^ cells (Supplementary Figure 1D), excluding a general growth defect for Pex5p^2D^ cells.

To assess if the Pex5p^2D^ mutant is impaired in its membrane binding ability, which would result in reduced matrix protein import, we prepared postnuclear supernatants of cells grown in oleic acid and expressing Pex5p^WT^, Pex5p^2A^ or Pex5p^2D^, separated them into a cytosolic fraction and an organellar pellet and analyzed the distribution of the different Pex5p variants along with cytoplasmic and membrane marker proteins (Supplementary Figure 1E). Quantitative analysis of immunoblot signals revealed no detectable difference between the Pex5p variants in their distribution between cytosolic fraction and organellar pellet, indicating that the phosphomimetic Pex5p variant is not affected in its general membrane binding ability.

To further assess the association of Pex5p variants with the peroxisomal docking complex, we affinity-purified TPA-tagged versions of Pex5p^WT^, Pex5p^2A^ and Pex5p^2D^ from digitonin-solubilized membrane fractions. Immunoblot analysis of the protein complexes using antibodies against Pex5p and components of the docking complex (Pex14p/Pex17p/Pex13p) revealed that the composition of the complex and the abundance of individual proteins in Pex5p complexes is comparable irrespective of the Pex5p variant expressed (Supplementary Figure 1F, top). This result was corroborated in reverse affinity-purification experiments using TPA-tagged Pex14p as bait (Supplementary Figure 1F, bottom). Thus, phosphosite mutations of S507/523 located in the TPR domain do not affect the association of Pex5p with the docking complex, but do affect the capacity to shuttle cargo into peroxisomes.

Taken together, our data indicate that phosphorylation of Pex5p at S507/523 reduces the import of peroxisomal matrix proteins by affecting a process occurring upstream of Pex5p binding to the import complex.

### Pex5p-S507/523D shows decreased cargo-binding affinity to Pcs60p

The TPR domain of Pex5p is essential for the binding of PTS1-proteins (Klein et al. 2001; Stanley et al. 2007). Thus, we hypothesized that phosphorylation of S507/523 in the TPR domain affects cargo binding and investigated changes in the direct interaction of Pex5p^2D^ and PTS1-protein compared to Pex5p^WT^. For this, we recombinantly expressed and purified Pex5p^WT^ and Pex5p^2D^ from *Escherichia coli.* Both forms were folded with no major structural changes, as measured by circular dichroism (Supplementary Figure 2A), and had a similar melting temperature of approximately 46 °C measured by circular dichroism coupled to thermal ramping and nano-differential scanning fluorimetry (Supplementary Figure 2B and C). Thus, our data indicate that Pex5p^2D^ exhibits no detectable changes in protein structure.

**Figure 2:**
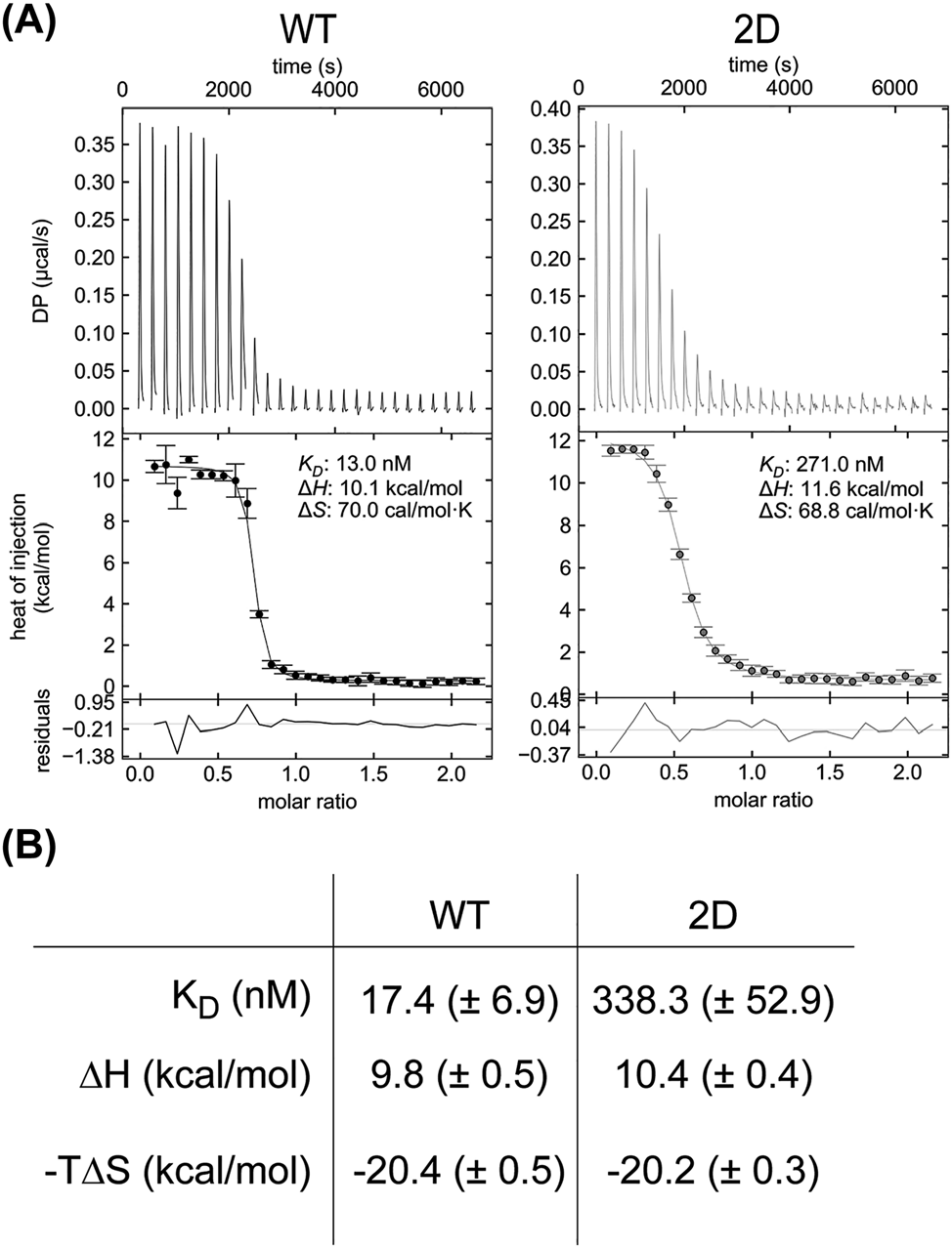
The Pex5p-S507/523D mutant shows lower affinity to cargo protein Pcs60p. (A) Representative isothermal titration calorimetry (ITC) raw data and integrated heat release for the interaction of Pcs60p with Pex5p^WT^ (left) or Pex5p^2D^ (right). DP, differential power. (B) Measurements by ITC of the equilibrium dissociation constants (K_*D*_) and enthalpy (Δ*H*) of the interaction between Pcs60p and Pex5p^WT^ or Pex5p^2D^ (*n* = 3, average ± standard deviation).

Next, we probed the interactions between Pex5p^WT^ or Pex5p^2D^ and the purified PTS1-protein Pcs60p using ITC (Figure 2A and Supplementary Figure 2D). Pcs60p is a peroxisomal oxalyl-CoA synthetase that is targeted to peroxisomes in a Pex5p-dependent manner (Blobel and Erdmann 1996) and its interaction with Pex5p has been previously studied (Hagen et al. 2015). Since Pcs60p has been shown to oligomerize at higher protein concentration (Hagen et al. 2015), we here employed an optimized protocol for measuring binding affinities by ITC using Pcs60p in lower concentration (100 µM instead of 400 µM). As a result, we measured an average equilibrium dissociation constant (K_*D*_) of 17.4 ± 6.9 nM between Pex5p^WT^ and Pcs60p (Figure 2A and B), which is approximately 10-fold lower than reported before (Hagen et al. 2015). We reason that the difference in our measured ITC binding data results from the formation of Pcs60p oligomers at high concentration, with parts of the oligomers being unable to act as binding partner for Pex5p. Strikingly, in comparison to Pex5p^WT^, we determined a considerably lower binding affinity between Pcs60p and phospho-mimicking Pex5p^2D^, with a K_*D*_ of 338.3 ± 52.9 nM (Figure 2B).

To conclude, phosphomimetic mutations at S507/523 in the second TPR triplet of Pex5p show no effect on receptor folding and stability, but strongly reduce the affinity of the receptor to its cargo, Pcs60p.

### Probing receptor-cargo complex characteristics by native MS

**Figure 3:**
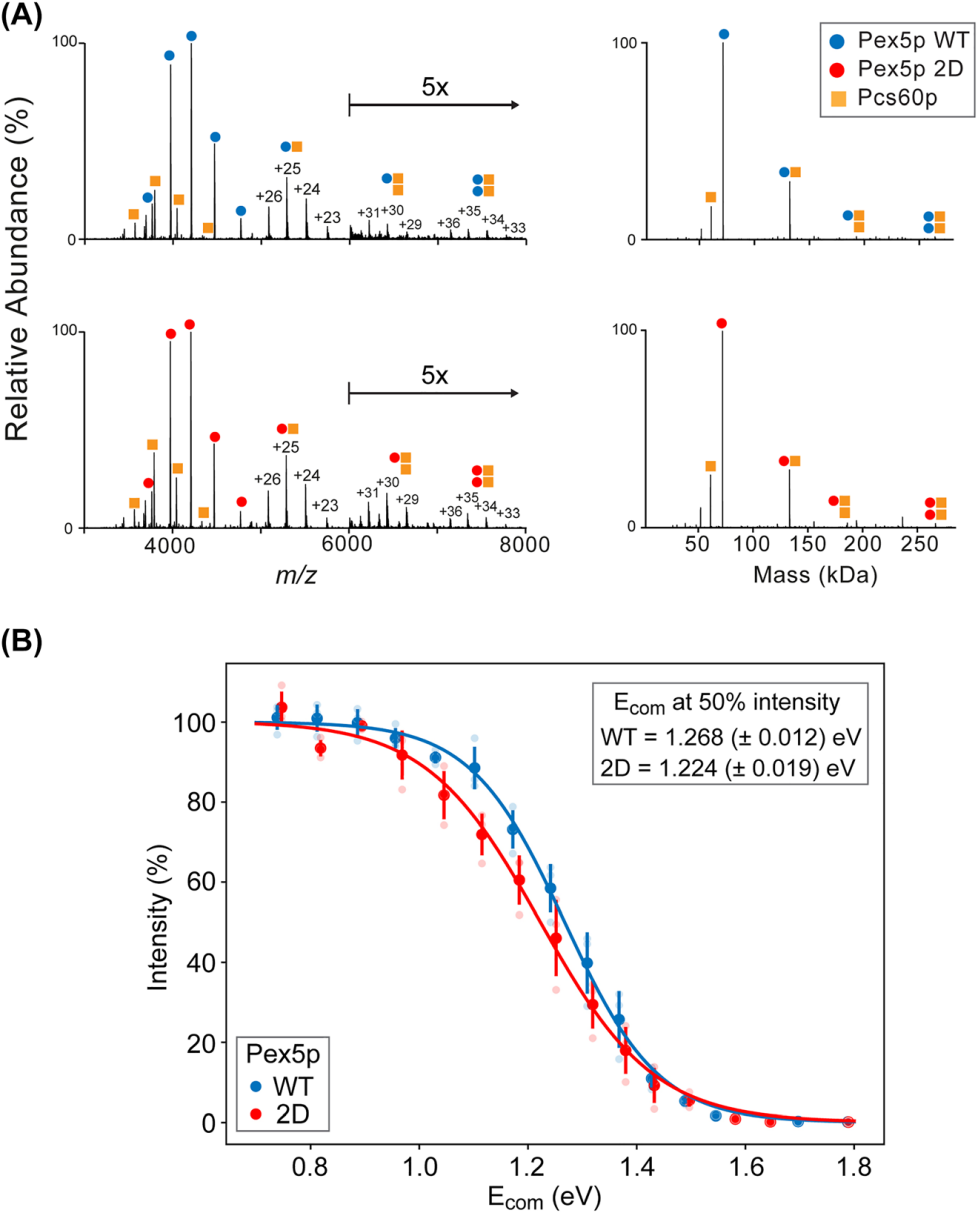
Native MS analysis of reconstituted Pex5p-Pcs60p complexes. (A) Native MS spectra of Pex5p^WT^ or Pex5p^2D^ (7.5 µM each) mixed with Pcs60p (5 µM). Spectra show ion series assigned to monomeric proteins and heteromeric complexes with annotated charge states of complexes (left) and the corresponding zero-charge mass spectra (right). Arrow, mass-to-charge (*m/z*) range with signal intensities magnified by 5-fold. Monomeric masses were assigned as follows: Pex5p^WT^, 71.459 kDa; Pex5p^2D^, 71.515 kDa; Pcs60p, 60.632 kDa (see Supplementary Figure 3A). Masses of heterodimeric receptor-cargo complexes were 132.091 kDa for Pex5p^WT^ and 132.147 kDa for Pex5p^2D^. Masses of low intensity 1:2 and 2:2 receptor-cargo complexes were 192.723 and 264.182 kDa for Pex5p^WT^ and 192.723 and 264.294 kDa for Pex5p^2D^. (B) Dissociation curves of heterodimeric Pex5p-Pcs60p complexes. For complex dissociation, the collision energy was gradually increased from 100 to 240 V in 10-V steps in native MS analysis (see Supplementary Figure 3B). The center-of-mass energy (*E*_com_) was calculated based on quantified ion series (charge states +23 to +26) of 1:1 complexes with Pex5p^WT^ (blue) and Pex5p^2D^ (red) for each acceleration voltage applied and plotted against summed intensities normalized to the total ion current of each spectrum. A Boltzmann function was used to fit sigmoidal curves to the mean of the dissociation data (*n* = 3). Error bars indicate standard deviation.

To study Pex5p-Pcs60p complexes in their near native and folded state in more detail, we employed native MS (Boeri Erba and Petosa 2015; Hernandez and Robinson 2007). We first determined the molecular masses and individual charge state distributions of purified Pex5p^WT^, Pex5p^2D^ and Pcs60p in native MS spectra (Supplementary Figure 3A). Both Pex5p^WT^ and Pex5p^2D^ were mainly detected in a monomeric state but also as low abundant dimers in the gas phase. In contrast, Pcs60p formed both monomers and dimers of nearly equal abundance, with additional low abundant tetramers and hexamers. We next reconstituted receptor-cargo complexes by incubating Pex5p^WT^ and Pex5p^2D^ with Pcs60p. Native MS analysis revealed a main complex population of 1:1 stoichiometry for either Pex5p^WT^ or Pex5p^2D^ with Pcs60p, whereas complexes with a 1:2 and 2:2 stoichiometry were of low abundance (Figure 3A).

To further probe the dissociation behavior of heterodimeric receptor-cargo complexes, we filtered out ion series of in solution-derived monomers (Pex5p variants, Pcs60p) and gradually increased the collisional energy. As expected, dissociation of these complexes occurred with a step-wise increase of collisional energy (Supplementary Figure 3B). We next compared the interaction stabilities of Pex5p^WT^ and Pex5p^2D^ with Pcs60p. For this, calculated center-of-mass energy (*E*_com_) values were plotted against normalized intensities of the 1:1 complexes and fitted to a Boltzmann function. Our data show that less energy is needed to dissociate 50% of gas phase Pex5p^2D^-Pcs60p complex compared to Pex5p^WT^-Pcs60p, indicating a difference in their binding affinities (Figure 3B). Thus, we inferred apparent gas phase dissociation constants (K_*D*_^*#*^) from our native MS data using a previously reported method (Danquah et al. 2019; Yefremova et al. 2017). Apparent Gibbs free energies calculated for each complex in the gas phase were plotted against the respective *E*_com_ values (Supplementary Figure 3C). By linear extrapolation to *E*_com_ = 0, we estimated apparent activation energies of complex dissociation that were translated into apparent *K*_*D*_^*#*^ values. The gas phase-derived dissociation constants of K_*D*_^*#*^ = 4.7 ± 1.8 for Pex5p^2D^ compared to K_*D*_^*#*^ = 0.45 ± 0.23 for Pex5p^WT^ indicate a 10-fold lower binding affinity between receptor and cargo upon the phosphomimetic mutation.

Taken together, native MS experiments revealed the formation of heterodimeric receptor-cargo complexes, with a decreased binding affinity of Pex5p^2D^ to Pcs60p compared to Pex5p^WT^ in the gas phase.

### Pex5p-S507/523D affects peroxisomal import of PTS1-proteins *in vivo*

**Figure 4:**
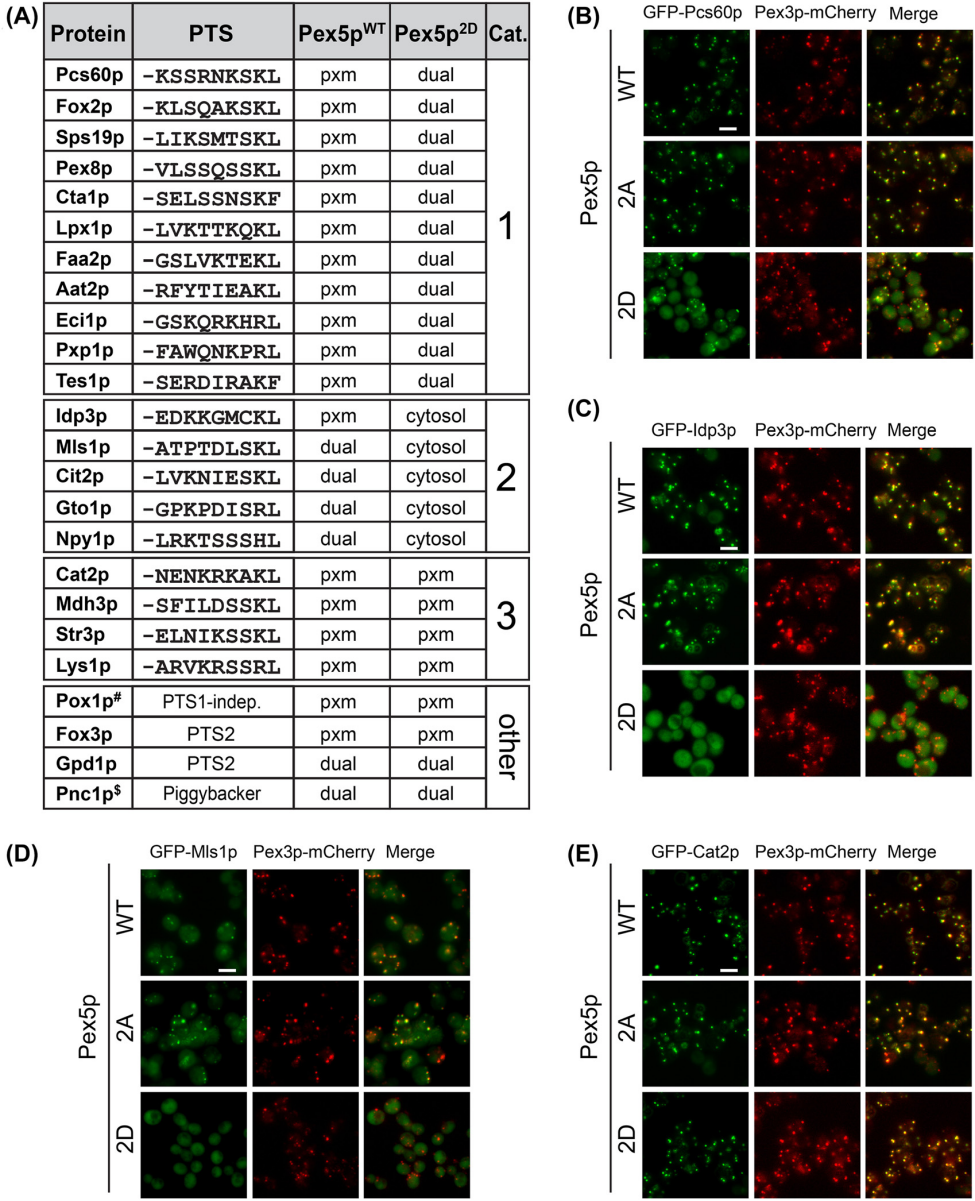
Expression of phospho-mimicking Pex5p^2D^ affects peroxisomal import of various, but not all, PTS1-proteins. (A) Overview of the subcellular distribution of 24 peroxisomal proteins following the PTS1 or PTS2 pathway in cells expressing Pex5p^WT^, Pex5p^2A^ or Pex5p^2D^. For fluorescence microscopy analysis, GFP-tagged versions of peroxisomal proteins were analyzed. For PTS1 proteins, the sequence of the carboxy-terminal PTS1 tripeptide and six amino acid residues adjacent to the PTS1 are shown. ^#^Pox1p is imported via Pex5p in a PTS1-independent manner (Klein et al. 2002); ^$^Pnc1p binds to Gpd1p and is imported via piggy-backing (Effelsberg et al. 2015). Category (Cat.) 1–3, moderate import phenotype (Cat. 1), strong import phenotype (Cat. 2), and no import phenotype (Cat. 3); PTS, peroxisomal targeting signal; pxm, peroxisomal localization; dual, peroxisomal and cytosolic localization. (B–E) Representative images of the subcellular distribution of GFP-Pcs60p (B), GFP-Idp3p (C), GFP-Mls1p (D), and GFP-Cat2p (E) in Pex5p^WT^-, Pex5p^2A^-, or Pex5p^2D^-expressing cells are shown. Pex3p-mCherry, peroxisomal marker protein; scale bar, 5 μm.

Our data show that the peroxisomal import of the artificial cargo protein GFP-SKL is decreased in cells expressing Pex5p^2D^ versus Pex5p^WT^ (Figure 1C) and that recombinant Pex5p^2D^ exhibits a lower binding affinity to its cargo protein Pcs60p compared to Pex5p^WT^ (Figures 2, 3B and Supplementary Figure 3C). However, it is not clear from both of these experiments whether this demonstrates a general reduction in the capacity of Pex5p to bind cargo, affecting the import of all cargos, or rather a way for modulating peroxisomal import that is dependent on the specific cargo chosen. To systematically observe the effect of the mutation on known Pex5p cargo proteins in an *in vivo* setting, we used a collection of yeast strains each expressing a peroxisomal protein amino-terminally tagged with GFP (Dahan et al. 2017; Yofe et al. 2016; Weill et al. 2018), as well as Pex3p-mCherry as a peroxisomal marker protein and either Pex5p^WT^, the Pex5p^2A^ or Pex5p^2D^ mutant integrated into the genome instead of the endogenous copy. Fluorescence microscopy was performed using a high-content screening platform (Breker et al. 2013) and the localization of GFP-tagged cargo proteins was assessed manually. We visualized a total of 20 known PTS1-proteins as well as matrix proteins that are imported via alternative pathways (e.g. PTS2 pathway, piggyback import) (Figure 4A). In accordance with our ITC and native MS data showing a reduced binding affinity between Pex5p^2D^ and Pcs60p compared to Pex5p^WT^ (Figures 2, 3B and Supplementary Figure 3C), GFP-Pcs60p exhibited an altered cellular distribution in Pex5p^2D^-expressing cells: while the protein was restricted to puncta co-localizing with Pex3p-mCherry in Pex5p^WT^ and Pex5p^2A^ cells, we observed both a punctate pattern and diffuse cytosolic fluorescence in Pex5p^2D^ cells, indicating its reduced peroxisomal import (Figure 4B).

The fluorescence microscopy experiment revealed that certain other PTS1-proteins exhibit a reduced peroxisomal import in Pex5p^2D^ cells as well (Figure 4A). We classified PTS1-proteins into three different categories according to the observed import phenotypes. Pcs60p (Figure 4B) and ten other PTS1-proteins showed a reduced peroxisomal import and constitute category 1 (Supplementary Figure 4A). In contrast, peroxisomal NADP-dependent isocitrate dehydrogenase (Idp3p; Figure 4C) and malate synthase 1 (Mls1p; Figure 4D) belong to category 2 characterized by no peroxisomal import (Supplementary Figure 4B). Interestingly, there were four PTS1-proteins without a noticeable effect on import in Pex5p^2D^ cells (category 3): carnitine acetyl-CoA transferase (Cat2p; Figure 4E), saccharopine dehydrogenase (Lys1p), peroxisomal malate dehydrogenase (Mdh3p) and peroxisomal cystathionine beta-lyase (Str3p) (Supplementary Figure 4C). We noticed that Cat2p and Lys1p exhibit an arginine at position −2 adjacent to the PTS1 tripeptide and this has been shown to be indicative for a strong interaction with Pex5p (Lametschwandtner et al. 1998). In Str3p, a lysine residue is found at position −2 and we hypothesize that this basic residue together with the adjacent SKL tripeptide increases the binding to Pex5p. For Mdh3, the unaffected targeting to peroxisomes cannot be explained by an adjacent basic residue to increase the binding affinity but rather by its high abundance (Rosenthal et al. 2020). In line with our findings, Cat2p, Mdh3p, and Lys1p were reported to have a high targeting priority to peroxisomes under both glucose and oleate condition (Rosenthal et al. 2020).

Among the proteins with reduced peroxisomal import in Pex5p^2D^ cells (category 1) is Pex8p, an essential component of the import machinery for peroxisomal matrix proteins and required for the biogenesis of functional peroxisomes (Agne et al. 2003; Rehling et al. 2000), which may raise the question if the reduced import of most PTS1 proteins in Pex5p^2D^ cells is a consequence of decreased peroxisomal Pex8p levels. Fluorescence microscopy analysis of GFP-tagged Mdh3p, a category 3 protein unaffected in Pex5p^2D^ cells, shows that its peroxisomal import generally depends on Pex8p: in *pex8*Δ cells, GFP-Mdh3p is mislocalized to the cytosol, exhibiting the same subcellular distribution as in cells lacking the PTS1 receptor Pex5p (Supplementary Figure 4D). This result is in agreement with a previous study reporting the mislocalization of Cta1p, Pcs60p and Fox3p to the cytosol in *pex8*Δ cells (Rehling et al. 2000) and consistent with the essential role of Pex8p for peroxisomal matrix protein import. However, the observation that GFP-Mdh3p shows wildtype-like peroxisomal import in Pex5p^2D^ cells despite reduced levels of Pex8p (as was also seen for Cat2p, Lys1p, and Str3p) indicates that the peroxisomal Pex8p fraction in the Pex5p^2D^ mutant is still sufficient for matrix protein import and that the import effects that we observe for the majority of the tested PTS1 proteins results from the S507/523D mutation in Pex5p.

The fact that some proteins showed no reduction in targeting demonstrates that the phospho-mimicking mutations in the *PEX5* gene did not simply result in a generally reduced-function allele affecting all PTS1 proteins and that phosphorylation is not just used to turn Pex5p activity ‘on’ or ‘off’. It rather shows that phosphorylation has a putative role in modulating substrate specificity and thereby presumably fine-tunes the import of PTS1-proteins into peroxisomes. The specificity of a reduced PTS1-protein import in cells expressing the phospho-mimicking Pex5p^2D^ mutant is demonstrated by our findings that neither Pox1p (fatty-acyl coenzyme A oxidase), which is imported via Pex5p in a PTS1-independent manner (Klein et al. 2002), nor the proteins Fox3p (3-ketoacyl-CoA thiolase) and Gpd1p (NAD-dependent glycerol-3-phosphate dehydrogenase), which both follow the PTS2 import pathway (Jung et al. 2010; Purdue et al. 1998), were affected in their peroxisomal import (Supplementary Figure 4E). Furthermore, we observed no differences in localization for the nicotinamidase Pnc1p, which is imported by piggy-backing on Gpd1p (Effelsberg et al. 2015) (Supplementary Figure 4E).

Taken together, our data reveal that peroxisomal import of a specific subset of PTS1 cargo proteins is affected by Pex5p phosphorylation at S507/523.

### Pex5p-S507/523D shows reduced binding to the carboxy-terminal region of PTS1-proteins

**Figure 5:**
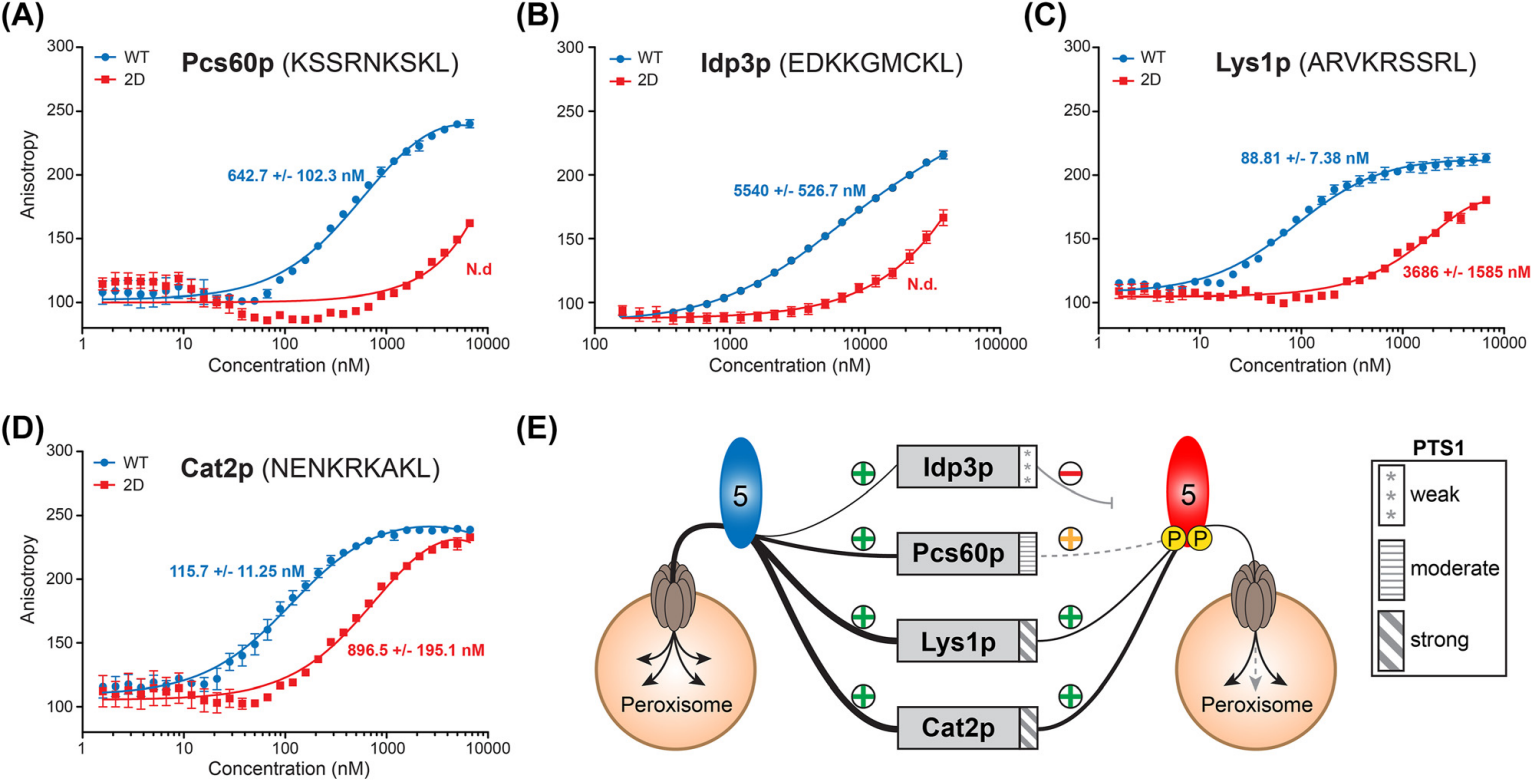
The phospho-mimicking S507/523D mutation alters the binding affinity of Pex5p to the carboxy-terminus of PTS1-proteins. The binding affinity of the extended PTS1 sequence of Pcs60p (A), Idp3p (B), Lys1p (C) and Cat2p (D) to Pex5p^WT^ and Pex5p^2D^ was analyzed by fluorescence anisotropy using fluorescently labeled peptides (10 nM) comprising the nine most carboxy-terminal amino acids of the respective PTS1-proteins and increasing concentrations of recombinant Pex5p variants (160 nM – 38 µM for measurements with the Idp3p peptide, 1.5 nM – 6.6 µM for all other measurements). K_*D*_ values were calculated by least square fitting of a binding-saturation model with one binding site. N.d., not determined; error bars indicate standard deviation (*n* = 2, Idp3p nonapeptide; *n* = 3, Lys1p, Cat2p and Pcs60p nonapeptides). (E) Model illustrating the differential effects of Pex5p-S507/523 phosphorylation on import of PTS1-proteins into peroxisomes. Proteins containing a high-affinity extended PTS1 sequence (e.g. Lys1p, Cat2p) bind to sufficient extent to phosphorylated Pex5p and are therefore efficiently imported into peroxisomes. In contrast, proteins containing a moderate- or low-affinity extended PTS1 sequence are imported to considerably lesser degree (e.g. Pcs60p) or not imported at all (e.g. Idp3p).

The consensus sequence of the PTS1 tripeptide has been defined as (S/A/H/C/E/P/Q/V) (K/R/H/Q) (L/F) (Notzel et al. 2016). In previous work, it has been shown that variants of this tripeptide exhibit different binding affinities to Pex5p (Lametschwandtner et al. 1998). Furthermore, amino acids upstream of the PTS1, referred to as extended PTS1, have also been shown to contribute to the binding to Pex5p (Brocard and Hartig 2006; DeLoache et al. 2016; Fodor et al. 2012; Hagen et al. 2015; Lametschwandtner et al. 1998; Rosenthal et al. 2020). To address whether the varying effect of the phosphomimetic mutant on the peroxisomal targeting of PTS1-proteins (Figure 4 and Supplementary Figure 4) relate to differences in the binding of the corresponding extended PTS1 sequences to Pex5p, we generated a set of fluorescently labeled peptides encompassing the nine carboxy-terminal residues of Pcs60p (moderate import phenotype, category 1) and Idp3p (strong import phenotype, category 2) as well as Lys1p and Cat2p (no import phenotype, category 3), and measured the *in vitro* binding affinities of Pex5p^2D^ and Pex5p^WT^ to these peptides by fluorescence anisotropy. For the Pcs60p nonapeptide KSSRNKSKL, we determined a moderate binding affinity to Pex5p^WT^, with a K_*D*_ of 642.7 ± 102.3 nM (Figure 5A). Notably, the measured binding affinity for the Pcs60p nonapeptide was lower compared to those for full-length Pcs60p (17.4 ± 6.9 nM) determined by ITC (Figure 2). A similar gain in binding affinity for full-length protein compared to a carboxy-terminal PTS1-peptide has been previously observed (Fodor et al. 2012; Stanley et al. 2006).

In comparison to the Pcs60p nonapeptide, the Idp3p nonapeptide EDKKGMCKL exhibited weaker binding to Pex5p^WT^ with a K_*D*_ of 5.54 ± 0.53 µM (Figure 5B). Interestingly, when using Pex5p^2D^, we could not measure binding saturation under the experimental condition applied, suggesting that binding of Pex5p^2D^ to both peptides is residual. Distinct from the binding data obtained for Pcs60p and Idp3p peptides, PTS1-nonapeptides of proteins not affected in their peroxisomal import (category 3; Figure 4A, E and Supplementary Figure 4C) exhibited a strong binding affinity for Pex5p^WT^, with a K_*D*_ of 88.81 ± 7.38 nM for the Lys1p peptide ARVKRSSRL and 115.7 ± 11.3 nM for the Cat2p peptide NENKRKAKL (Figure 5C and D), which is in line with recent findings (Rosenthal et al. 2020). Their binding affinity to Pex5p^2D^ was reduced, with a K_*D*_ of 3.69 µM for the Lys1p peptide and 896.5 nM for the Cat2p peptide, but this was now in the range where WT binds the low-affinity binders explaining why their targeting was not affected.

To conclude, the phosphorylation state of Pex5p at S507/523 in its TPR domain specifically affects the binding of PTS1-proteins within their carboxy-terminal region. In a simplified model (Figure 5E), we propose that for moderate or weak binders, this reduction in PTS1-dependent binding to phosphorylated Pex5p reduces or even prevents import into peroxisomes, as observed for Pcs60p and Idp3p, respectively (Figure 4B, C, 5A; Supplementary Figure 4A and B). However, for proteins containing high-affinity extended PTS1 sequences, as shown for Cat2p and Lys1p, binding to phosphorylated Pex5p is still sufficient for peroxisomal import and, thus, has little or no effect on their peroxisomal localization (Figures 4E, 5C, D and Supplementary Figure 4C).

## Discussion

Pex5p is the main peroxisomal import receptor for PTS1-proteins. Here, we show that Pex5p is phosphorylated at multiple sites *in vivo*. We identified and localized 22 phosphosites in Pex5p of *S. cerevisiae,* of which 14 were identified here for the first time, including pS507 and pS523 located in the TPR domain (Figure 1A).

Several serines that we identified to be phosphorylatable in Pex5p of *Saccharomyces cerevisae* Pex5p are conserved in the homologs of the methylotrophic yeast *Hansenula polymorpha*, human and/or mouse (in some cases featuring a threonine at the corresponding position in the sequence; Figure 1A). A Pex5p phosphosite conserved across all four species is S121, which is positioned within one of the WxxxF motifs involved in Pex14p binding at the peroxisomal membrane (Saidowsky et al. 2001; Otera et al. 2002). Interestingly, phosphorylation of the corresponding site in human PEX5, S141, plays a role in pexophagy (Zhang et al. 2015). Further conserved Pex5p phosphosites in the Pex14p-binding region and of so far unknown function are S118, S189, S192, and S255. Two phosphosites (pS7, pS25) are positioned adjacent to mono- and polyubiquitination sites of Pex5p from *S. cerevisiae*, which may indicate an interplay between these modifications for fine-tuning receptor recycling and/or removal.

An important structural region in Pex5p is comprised by its TPR domain that is directly involved in the binding of PTS1 cargo proteins (Klein et al. 2001; Otera et al. 2002; Stanley et al. 2007). We identified three phosphosites (pS330, pS507, pS523) in the TPR domain of Pex5p, with the first located in the first TPR triplet and the latter two in the second TPR triplet. Most interestingly, S507 is conserved in mammals and the neighboring residue of S523 is a phosphorylatable tyrosine in mammals (Figure 1A). Expressing the phospho-mimicking mutant Pex5p-S507/523D resulted in reduced import of GFP-SKL into peroxisomes (Figure 1C). This effect was neither observed for the single site mutants S507D and S523D (Figure 1C) nor for any other Pex5p site mutant investigated here (Supplementary Figure 1B). It suggests that the introduction of two negative charges (at position S507 and S523) in the TPR domain of Pex5p is required to affect cargo binding. Since Pex5p undergoes significant conformational changes upon ligand binding (Stanley et al. 2006), we hypothesize that the addition of negative charges on the surface of the TPR might alter the allosteric conformational changes mediated by PTS1 binding. This may likely affect how the PTS1 binding cavity tightens around a PTS1 peptide for high(er) affinity binding (Fodor et al. 2015). The observed decrease in PTS1-protein import capacity leads to an impaired growth of Pex5p^2D^-expressing cells in oleate (Figure 1D), which shows that this biochemical effect has physiological implications. Nonetheless, we note that Pex5p^2A^-expressing cells showed no import phenotype and grew similar to wildtype cells in oleate (Figure 1C and D). This might suggest that the Pex5p TPR phosphorylations are not physiologically relevant when cells metabolize oleate or rather have a modulatory effect for the targeting of different PTS1-proteins when peroxisomes start to proliferate or when other specific functions of peroxisomes than fatty acid beta-oxidation are needed. Since association of Pex5p^2D/2A^ mutants with the peroxisomal docking complex is not impaired (Supplementary Figure 1F), we focused on the effects of the phosphomutant to binding of PTS1 cargo proteins. *In vitro* measurements support a role for the phosphorylation sites in modulating this binding. First, ITC titration experiments showed a strongly reduced affinity for the binding of Pcs60p to Pex5p^2D^ compared to Pex5p^WT^, with a K_*D*_ increased by a factor of ∼20 (Figure 2A and B). Second, native MS analysis showed predominant formation of 1:1 Pex5p-Pcs60p complexes, and no differences in receptor-cargo stoichiometry was observed between Pex5p^WT^ and Pex5p^2D^ (Figure 3A). Third, gas-phase collisional activation revealed reduced stability of receptor-cargo complexes for Pex5p^2D^ (Figure 3B and Supplementary Figure 3B), with a 10-fold higher apparent K_*D*_^*#*^ compared to Pex5p^WT^ (Supplementary Figure 3C).

To explain the difference in K_*D*_ ratios obtained by ITC and native MS, we have to consider the experiment-specific conditions. The model we used to derive apparent dissociation constants in the gas phase (by applying in-solution thermodynamics) presumes a reaction governed by enthalpic interactions (Danquah et al. 2019; Yefremova et al. 2017). However, hydrophobic interactions, although partly preserved in the gas phase (Liu et al. 2009), are likely to be weakened by desolvation and loss of entropic contributions (Sharon and Robinson 2011). Importantly, although K_*D*_ values calculated by the two methods are different in their absolute values, both show that the binding affinity between Pcs60p and Pex5p^2D^ is significantly reduced by about an order of magnitude in comparison to Pex5p^WT^, which points to a phosphorylation-dependent mechanism for modulating receptor-cargo complex formation.

Our systematic *in vivo* analysis of Pex5p phosphorylation-dependent import of native peroxisomal proteins by fluorescence microscopy revealed that a specific set of PTS1-proteins exhibited moderate to strongly reduced import into peroxisomes in Pex5p^2D^-expressing cells, whereas peroxisomal import of Mdh3p, Lys1p, Cat2p and Str3p was not affected (Figure 4 and Supplementary Figure 4). Interestingly, these latter, unaffected peroxisomal enzymes have a protective role in oxidative stress response and intraperoxisomal redox balance (Al-Saryi et al. 2017; Franken et al. 2008; Higgins et al. 2002; O’Doherty et al. 2014). Moreover, Mdh3p, Lys1p, and Cat2p have recently been shown to be proteins with high targeting priority that are still imported into peroxisomes under conditions when the availability of Pex5p is limited (Rosenthal et al. 2020). Thus, it seems plausible that their function is continuously needed in peroxisomes. However, what are the specific features that determine whether the import of a PTS1-protein is affected by Pex5p phosphorylation in its TPR domain or not?

Cargo proteins bind to Pex5p via their PTS1, which canonically consists of the carboxy-terminal tripeptide SKL or variants thereof. In addition, the residues adjacent to the PTS1 tripeptide of cargo proteins constitute the extended PTS1 and markedly influence the strength of the interaction with the receptor (Brocard and Hartig 2006; DeLoache et al. 2016; Lametschwandtner et al. 1998; Rosenthal et al. 2020). Furthermore, for Lys1p and Cat2p it has recently been reported that the information for targeting priority is encoded in the last 10 amino acids (Rosenthal et al. 2020). In case of Pcs60p, the extended PTS1 has been demonstrated to indeed harbor a second binding site to Pex5p (Hagen et al. 2015). For Pex5p of *S. cerevisiae*, it has been shown that basic residues upstream of the consensus tripeptide SKL enhance PTS1-dependent protein import into peroxisomes (DeLoache et al. 2016; Lametschwandtner et al. 1998; Notzel et al. 2016). In particular, arginine at position −2 adjacent to the PTS1 tripeptide was found in PTS1-peptides strongly interacting with Pex5p, with a greater influence of upstream residues on binding for peptides that contain variants of the tripeptide SKL (Lametschwandtner et al. 1998). Based on the finding that a decrease in the binding affinity between PTS1-proteins and Pex5p reduces the efficiency of cargo import into peroxisomes, it has been proposed that the efficiency of peroxisomal matrix protein import mainly depends on the interaction strength of a cargo to its receptor Pex5p (Noguchi et al. 2013). We therefore considered that individual cargo proteins exhibit different binding affinities to Pex5p and that the extended PTS1 region (together with the specific PTS1 tripeptide) largely determines whether peroxisomal import of a cargo protein is affected by phosphorylation of Pex5p at S507/523 in its TPR domain or not.

In support of this notion, fluorescence anisotropy measurements using carboxy-terminal nonapeptides of differently affected PTS1-proteins revealed a correlation between binding affinities and import effect (Figures 4 and 5, Supplementary Figure 4). Thus, the strong import phenotype of Idp3p correlates well with a weak binding affinity of its nonapeptide EDKKGMCKL to Pex5p^WT^, whereas Cat2p and Lys1p, which display no import phenotype, feature nonapeptides with high binding affinities to Pex5p^WT^. Furthermore, although nonapeptides of Cat2p (NENKRKAKL) and Lys1p (ARVKRSSRL) exhibit reduced binding affinities to Pex5p^2D^, receptor-cargo binding appears to be sufficient for peroxisomal import of Cat2p and Lys1p in Pex5p^2D^-expressing cells. Cat2p and Lys1p both feature a carboxy-terminal sequence with a positively charged arginine in the −2 position of the PTS1 tripeptide, which is not the case for the Idp3p. For the Pcs60p nonapeptide (KSSRNKSKL), we measured a moderate binding affinity to Pex5p^WT^, which correlates with the moderate import phenotype of Pcs60p in Pex5p^2D^-expressing cells. Thus, phosphorylation of Pex5p at S507/523 generally affects binding to Pex5p but whether this results in reduced import or not depends on the residual binding affinity to Pex5p. In case of a cargo protein with a high-affinity extended PTS1 sequence (e.g. Cat2p, Lys1p), the decreased affinity is still sufficient to promote peroxisomal targeting. For proteins with a moderate (e.g. Pcs60p) or low-affinity (e.g. Idp3p) extended PTS1 sequence, this results in a considerably reduced or no peroxisomal import, respectively (Figures 4 and 5, Supplementary Figure 4). Hence, proteins may have evolved to have specific affinities to Pex5p to support essential peroxisomal functions under conditions when general import capacity is downregulated or under specific conditions when Pex5p becomes phosphorylated in its TPR domain.

Taken together, we report a mechanism of posttranslational regulation for the PTS1-dependent import of peroxisomal proteins via Pex5p. We show that phosphorylation of Pex5p at S507/523 provides a means by which targeting of a specific set of PTS1-proteins to peroxisomes is sustained, whereas simultaneously peroxisomal import of other PTS1-proteins is decreased or prevented. This difference in import phenotypes can be explained by the presence of variable-affinity PTS1 sequences affecting the extent of Pex5p binding. Thus, our findings support the view that protein import into peroxisomes is a highly dynamic process, which can be specifically modulated by the phosphorylation state of Pex5p. We hypothesize that Pex5p-S507/523 phosphorylation is a transient process that allows the cells to rapidly adjust the flux, and potentially composition, of matrix proteins into peroxisomes during changing environmental or metabolic requirements. We identified these Pex5p phosphorylations in cells grown in oleate, i.e. under peroxisome proliferating conditions. In the future, it will be of interest to identify conditions under which a switching between the phosphorylation and dephosphorylation state of Pex5p-S507/523 occurs and what the kinase(s) and phosphatase(s) regulating these events are. A better understanding of how reducing the import of most, while at the same time sustaining the import of few proteins is of advantage for cellular growth and viability will inform us of the metabolic rewiring that peroxisomes can undergo to respond to changing metabolic needs.

## Materials and methods

### Plasmids and cloning techniques

Plasmids used in this study as well as primers, restriction enzymes, DNA templates and target plasmids are listed in Supplementary Table 2; sequences of the primers are provided in Supplementary Table 3. Amplification of plasmids was performed in *E. coli* strains DH5α according to standard procedures. Plasmids were generated using standard cloning techniques (Sambrook et al. 1989). DNA manipulations resulting in the exchange of selected single serine residues in Pex5p to alanine or aspartate were performed by site-directed mutagenesis (Papworth et al. 1996). For the simultaneous introduction of multiple mutations into the *PEX5* gene, custom-made double-strand DNA fragments were obtained from Thermo Fisher Scientific GeneArt (Germany) and subsequently inserted into PEX5 using restriction enzymes. Sequences of GeneArt constructs are provided in Supplementary Table 4. For the use of plasmids in high-content fluorescence microscopy screens, the URA3 marker present in the pRS416 backbone was exchanged with a KanMX resistance cassete by Gibson assembly (Gibson et al. 2009). For recombinant expression of N-terminally His-tagged Pex5p wildtype and phosphosite mutants, the pET9d vector containing the 6His-PEX5 sequence was used (pSH462) (Kerssen et al. 2006) and phosphosite mutants were generated as described above.

### Sequence alignment

A multiple protein sequence alignment of Pex5p from *S. cerevisiae* (UniProtKB ID P35056) with its homologs in *H. polymorpha* (Q01495), *Homo sapiens* (P50542) and *Mus musculus* (O09012) was performed with ClustalOmega (Madeira et al. 2019).

### Strains, media, and growth conditions

*S. cerevisiae* strains used in this study are listed in Supplementary Table 5 as well as information about primers and DNA templates used for genomic manipulation of the target strain. The sequences of the primers used to amplify integration cassettes are listed in Supplementary Table 3. For the generation of SC38, gene disruption using pUG27 and pUG73 and marker rescue using pSH47 (containing Cre recombinase for LoxP recombination) were performed as described (Gueldener et al. 1996). Deletion of the *PEX5* gene was introduced according to (Goldstein and McCusker 1999).

Synthetic complete (SC) medium consisted of 0.17% (w/v) yeast nitrogen base (YNB) without amino acids, 0.5% (w/v) ammonium sulfate, and 0.3% (w/v) glucose (pH 6.0) supplemented with appropriate amino acids (Schummer et al. 2017). YNO medium contained 0.1% (v/v) oleic acid, 0.05% (v/v) Tween 40, 0.5% (w/v) ammonium sulfate, and 0.17% (w/v) yeast nitrogen base without amino acids, adjusted to pH 6.0 and supplemented with appropriate amino acids. For growth under peroxisome-proliferating conditions, cells were cultured at 30 °C in SC medium until they reached an optical density at 600 nm (OD_600_) of 1–1.5 and then shifted to YNO medium to induce peroxisome proliferation. Cells were grown for further 12–16 h or as indicated. For growth of cells in glucose medium, SC medium was supplemented with 2% (w/v) glucose.

### Yeast cell fractionation

Spheroplasting of yeast cells, homogenization, preparation of postnuclear supernatants and generation of cytosolic fractions and organellar pellets by centrifugation (25,000 *g* for 15 min at 4 °C) were performed as described previously (Erdmann et al. 1989).

### Whole yeast cell lysates and cytosol/membrane separation

Yeast cells were harvested by centrifugation and washed twice with deionized water. Whole cell lysates were prepared by TCA precipitation as described before (Platta et al. 2004). Precipitated proteins were collected by centrifugation (10 min, 21,000 *g*), resuspended in 1% (w/v) SDS/0.1 M NaOH and analyzed by SDS-PAGE according to standard protocols. For cytosol/membrane separation, yeast cells were resuspended in lysis buffer containing protease and phosphatase inhibitors (20 mM Tris, 80 mM NaCl, pH 7.5, 174 μg ml^−1^ PMSF, 2 μg ml^−1^ aprotinin, 0.35 μg ml^−1^ bestatin, 1 μg ml^−1^ pepstatin, 2.5 μg ml^−1^ leupeptin, 160 μg ml^−1^ benzamidine, 5 μg ml^−1^ antipain, 6 μg ml^−1^ chymostatin, 0.4 mg ml^−1^ NaF, 368 μg ml^−1^ sodium orthovanadate, 2.16 mg ml^−1^ β-glycerolphosphate). Lysates were prepared by breaking the cells with glass beads (Lamb et al. 1994). Cell debris was removed followed by centrifugation of the lysate at 100,000 *g* for 90 min at 4 °C to separate the crude membrane from the soluble, cytosolic fraction. Protein concentrations were determined using the Bradford assay and bovine serum albumin as standard (Bradford 1976).

### Affinity purification of TPA-tagged Pex5p and Pex14p

Pex5p was affinity-purified from crude membrane and cytosolic fractions prepared from oleic acid-induced cells expressing TPA-tagged Pex5p as described before with slight modifications (Agne et al. 2003; Schafer et al. 2004). In brief, cytosolic and membrane fractions were prepared as described above using lysis buffer containing 10% (v/v) glycerol. For the analysis of Pex5p phosphorylation sites, crude membrane fractions were resuspended in glycerol-containing lysis buffer, the protein concentration was adjusted to 5 mg ml^−1^, and proteins were solubilized using 1% (v/v) Triton X-100. Unsolubilized material was removed by centrifugation (100,000 *g*, 1 h, 4 °C). The cleared detergent extracts as well as cytosolic fractions were subjected to affinity purification via IgG Sepharose. Proteins bound to the matrix were eluted by incubation with AcTEV protease (Thermo Fisher Scientific/Invitrogen) followed by addition of Ni-NTA agarose to remove the His-tagged TEV protease. Eluted proteins were collected by centrifugation and precipitated by adding the fivefold volume of ice-cold acetone followed by incubation at −20 °C for at least 1 h.

Native Pex5p^TPA^ and Pex14p^TPA^ complexes were purified from crude membrane fractions as described above except that the protein concentration of the resuspended crude membrane fraction was adjusted to 3.5 mg ml^−1^ and that 1% (w/v) digitonin was used for solubilization.

### Sample preparation for fluorescence microscopy

Cells expressing distinct Pex5p variants (wildtype, Pex5p^2A^ or Pex5p^2D^) and GFP-SKL were grown overnight in 50 ml YNO medium supplemented with 0.1% (w/v) glucose to an OD_600_ of 1–1.5. Aliquots of 5.4 ml were taken and formaldehyde was added to a final concentration of 3.7% (v/v). Samples were incubated for 10 min at RT with slight agitation. Cells were collected by centrifugation (5 min at 2000 *g*), resuspended in 1 ml of 0.1 M potassium phosphate buffer (pH 6.8) containing 3.7% (v/v) formaldehyde and incubated for 1 h at RT with slight agitation. Following centrifugation (30 s at 16,000 *g*), cells were resuspended in 1 ml of 0.1 M potassium phosphate buffer (pH 6.8) containing 10 mM ethanolamine and incubated for further 10 min at RT. Subsequently, cells were washed twice with phosphate-buffered saline (PBS) and resuspended in 10–20 µl of PBS containing 0.1% (v/v) Triton X-100. Fixed cells were analyzed within 48 h after preparation.

### Fluorescence microscopy of cells expressing GFP-SKL

Fluorescence microscopy was performed using a Zeiss Axio Observer.Z1 microscope equipped with an alpha Plan-Aprochromat 100× objective and an AxioCam MR R3. GFP signal was visualized with a 38 HE filterset from Zeiss. Images were processed using Adobe Photoshop CS5 (version 12.0.4 ×64; Adobe Systems Incorporated). To document the structural integrity of cells, bright field images were acquired, adjusted in a way that only the cell boundaries were visible and subsequently added into the blue channel in Photoshop as reported before (Motley and Hettema 2007).

### Immunoblotting and antibodies

Immunoblot analyses were performed according to standard procedures. Polyclonal rabbit antibodies used were raised against Pex5p (Albertini et al. 1997), Pex14p (Girzalsky et al. 1999), Pex13p (Girzalsky et al. 1999) and Pex17p (Huhse et al. 1998). To detect GFP, monoclonal mouse antibodies (Sigma-Aldrich/Merck or Roche Diagnostics, Germany) were used. Immuno-reactive complexes were visualized either using the IRDye 800CW goat anti-mouse IgG or IRDye 680RD goat anti-rabbit secondary antibody (Li-COR Biosciences, Bad Homburg, Germany) followed by detection with an ‘Infrarot Imaging System’ (Li-COR Biosciences) or using horseradish-peroxidase coupled anti-rabbit or anti-mouse antibodies (Sigma-Aldrich/Merck, Germany) and subsequent detection of chemiluminescence signals with a ChemoCam Camera system (INTAS Science instruments GmbH, Göttingen, Germany). Quantification of immunoblot signals (Supplementary Figure 1E; *n* = 3) was performed using the software Image Studio Lite (LI-COR Biosciences).

### Expression and purification of recombinant Pex5p and Pcs60p

Recombinant His_6_-tagged variants of Pex5p (^His^Pex5p; wildtype and phosphosite mutant) or Pcs60p, N-terminally linked to a GST-tag with a thrombin cleavage site, were expressed in *E. coli* BL21 RIL (DE3) cells (New England Biolabs GmbH) and purified via their respective affinity tag. Cells transformed with pSH462 (Pex5p^WT^), pSF482 (Pex5p^2D^) or pSH490 (Pcs60p) were grown overnight at 37 °C in LB medium [1% (w/v) peptone, 0.5% (w/v) yeast extract, and 0.5% (w/v) NaCl] containing 50 μg ml^−1^ kanamycin (Pex5pWT and 2D mutant) or 50 μg ml^−1^ ampicillin (Pcs60p) and 25 μg ml^−1^ chloramphenicol. Autoinduction medium (Studier 2005) [25 mM Na_2_HPO_4_, 25 mM KH_2_PO_4_, 50 mM NH_4_Cl, 5 mM Na_2_SO_4_, 2 mM MgSO_4_, 0.05% (w/v) glucose, 0.2% (w/v) lactose, 0.5% (w/v) glycerol, 0.1% (w/v) trypton/peptone, 0.05% (w/v) yeast extract, 50 mM FeCl_3_, 20 mM CaCl_2_, 10 mM MnCl_2_, 10 mM ZnCl_2_, 2 mM CoCl_2_, 2 mM CuCl_2_, 2 mM NiCl_2_, 2 mM NaMoO_4_, 2 mM Na_2_SeO_3_, 2 mM H_3_BO_3_] with appropriate antibiotics was inoculated with a 1:50 dilution of an overnight culture and incubated for 20–24 h at 24 °C. Cells were harvested by centrifugation (10 min at 7000 *g*) and resuspended in either lysis buffer 1 [50 mM HEPES, 0.15 M NaCl, 5% (v/v) glycerol, 0.2 mM TCEP, 10 mM imidazole pH 7.5; 5 ml/g cells] for cells expressing ^His^Pex5p proteins or lysis buffer 2 (22 mM Na_2_HPO_4_, 2.8 mM NaHPO_4_, 150 mM NaCl, pH 7.5) for ^GST^Pcs60p-expressing cells. Lysis buffers were supplemented with protease inhibitors as described above for the cytosol/membrane separation of yeast cells (lysis buffer 2 without NaF and benzamiidine to allow for later thrombin cleavage) and 1 mg ml^−1^ lysozyme. Following incubation on ice for 1 h, 10 mM MgCl_2_ (final concentration) and 50 units benzonase were added and cells were incubated for further 15 min on ice. Cells were lysed using an EmulsiFlex C5 homogenizer (Avestin Europe GmbH, Mannheim, Germany) applying three iterations. Cell debris was removed by centrifugation (25,000 *g*, 40 min, 4 °C) and the supernatant was filtered through 0.22 µm membranes. All following steps were performed at 4 °C and an ÄKTA Pure FPLC system (GE Healthcare) was used for chromatographic separations. Cleared lysates containing ^His^Pex5p proteins were loaded onto a Ni-NTA column (HisTrap HP, 1 ml; GE Healthcare). The column was washed with 5 column volumes of wash buffer [50 mM HEPES, 0.5 M NaCl, 5% (v/v) glycerol, 0.2 mM TCEP, 20 mM imidazole pH 7.5] followed by 3 × 10 min on-column incubation with 5 mM ATP and 10 mM MgCl_2_ (in wash buffer) to remove bacterial DnaK (Rial and Ceccarelli 2002). Proteins were eluted with 10 column volumes of elution buffer (50 mM HEPES, 0.15 M NaCl, 0.2 mM TCEP, 300 mM imidazole pH 7.5). The elution of proteins was monitored at 280 nm. Peak fractions were pooled and concentrated to <1 ml using centrifugal filter units with a molecular weight cut-off of 10K (Amicon Ultra 2 ml, Merck Millipore). Concentrated eluates were dialyzed overnight against 50 mM HEPES (pH7.5). Proteins were further purified by ion exchange chromatography using a MonoQ 5/50 GL anion-exchange column (GE Healthcare) and applying a salt gradient ranging from 100 to 500 mM NaCl in 50 mM HEPES (pH 7.5). Fractions containing ^His^Pex5p, which elutes at a conductivity of approx. 24 mS/cm, were pooled, concentrated to about 600 µl and applied onto a Superdex 200 increase 10/300 GL column for size-exclusion chromatography using SEC buffer (50 mM HEPES, 150 mM NaCl, pH 7.5). For the purification of Pcs60p, lysates were loaded onto a GST-column (GSTrap, 1 ml; GE Healthcare), which was subsequently washed with 5 column volumes of lysis buffer 2. Peak fractions were concentrated to about 1 ml and incubated overnight with 80 U of thrombin (Serva) on a rotating wheel to cleave the GST tag. Further purification of Pcs60p and removal of thrombin were achieved by SEC as described for ^His^Pex5p.

### Mass spectrometric analysis of phosphorylated peptides

For the analysis of Pex5p *in vivo* phosphosites, affinity-purified and acetone-precipitated Pex5p from cytosolic and membrane fractions was resuspended in 100 µl of 60% (v/v) methanol/20 mM ammonium bicarbonate. Cysteine residues were reduced with 5 mM Tris(2-carboxy-ethyl)phosphine (30 min at 37 °C) and subsequently alkylated with 50 mM iodoacetamide (30 min at RT in the dark), followed by protein digestion with trypsin (Promega; 375 ng trypsin per sample, 4 h at 42 °C). Phosphopeptides were enriched from tryptic digests using titanium dioxide (TiO_2_, GL Sciences Incorporated, cat. # 5020–75000). All steps were performed at 4 °C. TiO_2_ beads were prepared as slurry in 100% acetonitrile (ACN) at a ratio of 1:2, washed twice with washing buffer [80% (v/v) ACN/0.1% (v/v) trifluoroacetic acid (TFA)] and dissolved in 1× loading buffer (see below). For each enrichment, 15 µl of TiO_2_ slurry were used. Proteolytic peptides were diluted by adding the same volume of 2× loading buffer [40% (v/v) acetic acid, 40 mg ml^−1^ dihydroxybenzoic acid, 840 mM 1-octansulfonic acid, 0.2% (v/v) heptafluorobutyric acid], added to 15 µl TiO_2_ slurry and incubated for 20 min on a rotating wheel. Beads were washed twice with washing buffer. 50 µl of elution buffer (50 mM ammonium dihydrogenphosphate, pH 10.5) were added and the beads were incubated for further 10 min on a rotating wheel. Eluted phosphopeptides were collected by centrifugation (2500 *g*, 2 min) through StageTips prepared with three layers of Empore Octyl C8 extraction disc material (Supeclo, Bellefonte, USA) into glass vials and acidified by adding 8 µl of 100% TFA. Peptide mixtures were dried *in vacuo* and resuspended in 0.1% TFA prior to liquid chromatography (LC)-MS analysis.

LC-MS analyses were performed using the UltiMate 3000 RSLCnano HPLC system (Thermo Fisher Scientific, Dreieich, Germany) directly coupled to a Q Exactive plus instrument (Thermo Fisher Scientific, Bremen, Germany). The RSLC system was equipped with 5 × 0.3 mm PepMap™ C18 μ-precolumns (Thermo Fisher Scientific, Bremen, Germany) for washing and preconcentration of the peptide mixtures and 50 cm × 75 μm C18 reversed-phase nano LC columns (Acclaim PepMap™ RSLC column; 2 μm particle size; 100 Å pore size; Thermo Scientific) for peptide separation at a temperature of 40 °C. Peptides were separated using a binary solvent system consisting of 0.1% (v/v) formic acid (solvent A) and 86% (v/v) ACN/0.1% (v/v) formic acid (solvent B) at a flow rate of 250 nl min^−1^. Peptides were eluted with a gradient of 3–40% solvent B in 30 min followed by 40–90% B in 5 min and 5 min at 95% B.

The Q Exactive Plus mass spectrometer was equipped with a Nanospray Flex ion source and stainless steel emitters (Thermo Scientific) and externally calibrated using standard compounds. Full MS scans in the range of *m/z* 375–1700 were acquired at a resolution of 70,000 (at *m/z* 200) with an automatic gain control (AGC) of 3 × 10^6^ ions and a maximum fill time of 60 ms. Up to 12 of the most intense multiply charged precursor ions were selected for fragmentation by higher-energy collisional dissociation at a normalized collision energy of 28%. Fragment spectra were acquired at a resolution of 35,000, a signal threshold of 5800, an AGC of 1 × 10^5^ ions, and a maximum fill time of 120 ms. The dynamic exclusion time for previously fragmented precursor ions was set to 45 s.

Mass spectrometric raw data were searched against a yeast proteome dataset downloaded from the *Saccharomyces* genome database (SGD), in which Pex5p was C-terminally modified with appropriate amino acid residues after TEV cleavage of the TPA tag, using MaxQuant/Andromeda (Cox and Mann 2008; Cox et al. 2011) (v. 1.5.5.1) with default settings. Oxidation of methionine, N-terminal acetylation and phosphorylation of serine, threonine and tyrosine residues were selected as variable modifications, carbamidomethylation of cysteine was set as fixed modification. Mass tolerances for precursor and fragment ions were 4.5 and 20 ppm, respectively. The option ‘match between runs’ was disabled. Skyline (MacLean et al. 2010) (v. 3.6.0.10163) was used to manually verify the correct assignment of phosphosites. Pex5p *in vivo* phosphosites reported in this study were identified by MS/MS in at least two out of three biological replicates in cytosolic or membrane fractions with a mass error tolerance of 5 ppm. In addition, the corresponding isotopic pattern of a phosphopeptide ion was required to comprise at least three isotopic peaks.

### Native mass spectrometric analyses

Recombinantly expressed Pex5p variants and Pcs60p protein solutions were buffer-exchanged into 200 mM ammonium acetate pH 6.8 at 4 °C using microcentrifuge gel filtration columns Bio-Spin 6 (Bio-Rad Laboratories). Protein concentration was determined spectrophotometrically using a Nanodrop 2000c (Thermo Scientific) by measuring absorption at 280 nm. Proteins were either analyzed individually or as mixture of Pex5p variant with Pcs60p at indicated concentrations to study receptor-cargo complex formation. For this, Pex5p^WT^ or Pex5p^2D^ mutant was incubated with Pcs60p for 5 min on ice. Spectra were obtained using a Synapt HDMS (Waters and MS Vision) equipped with an *m/z* 32,000 range quadrupole by introducing 3 µl sample with an in-house manufactured gold-coated capillary needle (Brosilicate Thin Wall with Filament Clark Capillary Glass, OD 1.0 mm, ID 0.78 mm; Harvard Apparatus). Capillary voltage was set to 1.3 kV and cone voltage to 180 V. The gas inlet of the three consecutive traveling wave cells, referred to as trap, IMS and transfer cell, was modified by additional needle valves to individually adjust the flow of the collision gas to the trap and transfer cell and, optionally, to the IMS cell. Trap and IMS cells were used for collision-induced dissociation with argon gas at a flow rate of 1 ml min^−1^. Similar acceleration voltages were applied to the trap and the IMS cells (trap DC bias) and the sum of both is given as total collision energy. The transfer cell was generally kept at a pressure of about 20% of that in the trap cell and a low acceleration voltage of 5 V. For measurements on single proteins as well as for Pex5p-Pcs60p titration experiments, the quad profile was set to 80% scan time ranging from *m/z* 2000 to 5000 with a 10% dwell time at each *m/z* 2000 and 5000 and the collision energy was set to 100 V. For receptor-cargo stability experiments, we used 7.5 µM Pex5p^WT^ or Pex5p^2D^ together with 5 µM Pcs60p as most suitable for 1:1 complex formation. For more sensitive and accurate signal detection of the 1:1 complex and its products resulting from gas-phase decomposition, we filtered out ion series of in solution-derived monomers (Pex5p variants, Pcs60p) using the quadrupole mass filter profile to impose a low mass cut-off. The quad profile was fixed at *m/z* 5800 with collision energies ranging from 100 to 240 V increasing in 10 V steps.

Spectra were calibrated externally using cesium iodide. For spectra analysis and processing, MassLynx v 4.1 (Waters) and UniDec v 2.7.3 (Marty et al. 2015) were used. Quantification of complex abundances was performed based on area under signals using a similar approach as previously described (Danquah et al. 2019; Yefremova et al. 2017). To compare stabilities of receptor-cargo complexes for Pex5p^WT^ and Pex5p^2D^ the collisional activation was quantified by calculating center-of-mass energies (*E*_com_) based on the abundance-weighted mean charge states of the 1:1 complex for each acceleration voltage. Charge states ranging from +23 to +26 were included as they gave rise to signals with highest intensities that were clearly detectable. Normalized intensities were plotted against *E*_com_ and fitted to a Boltzmann sigmoidal equation using the Levenberg-Marquardt nonlinear least-squares fitting algorithm in the Python module SciPy (Virtanen et al. 2020). Values for *E*_com_ at 50% intensity representing the energy needed to dissociate half of the complexes were obtained as fitted parameters for the inflection point of the curve. Confidence intervals within one standard deviation (*p* = 0.68) were derived by computing the upper quantile of an F-statistic as described before (Beechem 1992). To this end, parameters were systematically altered over a range of values while determining whether the mean square deviation of a fitted curve at each value was significantly different from the mean square deviation at the optimum value.

### K^#^_*D*_ approximation using native MS

Native MS and collisional activation were used to probe complex stabilities and approximate apparent dissociation constants (K_*D*_^#^) of heterodimeric complexes in the gas-phase, based on basic considerations as described before (Yefremova et al. 2017). Normalized signal intensities of the observed 1:1 complexes were used to derive apparent Gibbs free energies of the gas phase dissociation:

$$\frac{\Delta G^{\#}}{T}=-R*\ln\left(\frac{100\%-\text{norm}.\text{Intensity}}{\text{norm}.\text{Intensity}}\right)$$

Here, *R* is the gas constant and *T* the absolute temperature, which is not known for our gas phase conditions. Center-of-mass collision energies *E*_com_ were calculated as described taking into account the intensity weighted mean charge state of the ion series recorded in native MS spectra of the complex, which was shown to be a valid representation of individual charge states (Danquah et al. 2019; Yefremova et al. 2017). Gibbs free energies divided by the absolute temperature (Danquah et al. 2019) were then plotted against center-of-mass collision energies in a linear free energy approximation (Yefremova et al. 2017):

$$\frac{\Delta G^{\#}}{T}=\frac{\Delta G_{0}^{\#}}{T}-n*E_{\text{com}}$$

with Δ*G*_0_^#^/T and *n* representing intercept and slope of the linear relationship, respectively. Extrapolation to *E*_com_ = 0 yields the apparent activation energy of complex dissociation at ‘zero’ external activation. The dimensionless apparent gas phase dissociation constants were calculated as

$${\text{K}}_{D}^{\#}=e^{\left(\frac{\Delta G_{0}^{\#}}{-RT}\right)}$$

### Isothermal titration calorimetry

All proteins were dialyzed against 50 mM HEPES, pH 7.5, 150 mM NaCl, diluted, filtered, and degassed shortly before performing measurements. ITC measurements were conducted at 25 °C on a MicroCal VP-ITC with 10 μM full-length Pex5p^WT^ and Pex5p^2D^ mutant as a sample in the cell and 100 μM wildtype Pcs60p as a titration ligand. Pcs60p constructs were injected in volumes of 10 μl each in a total of 30 steps, resulting in a 2-fold excess of Pcs60p at the end of each titration experiment. Thermograms were integrated by NITPIC (Keller et al. 2012), data were fitted using SEDPHAT (Zhao et al. 2015) and plotted with GUSSI (Brautigam et al. 2016).

### Circular dichroism

Circular dichroism (CD) experiments were performed on a J-810 spectropolarimeter (Jasco). Proteins were dialyzed against 25 mM phosphate buffer (pH 7.5), 150 mM NaF. Far-UV spectra were recorded between 180 and 280 nm, using a 1-mm cuvette and a concentration of 0.2 mg ml^−1^ protein. The machine settings were 1 nm bandwidth, 1 s response, 1 nm data pitch and 100 nm min^−1^ scan speed.

Thermal unfolding coupled to CD was performed between 20 °C and 70 °C with a measure in triplicate every 5 °C increment. Millidegree values at 220 nm were plotted against their temperature and the melting temperature calculated using a Boltzmann sigmoidal equation.

### Nano differential scanning fluorimetry

Melting temperatures were measured by Nano differential scanning fluorimetry using a Prometheus NT.48 (Nanotemper). Tryptophan intrinsic fluorescence emission was recorded at 330 and 350 nm with 30% excitation power during thermal ramping from 20 to 90 °C (1 °C min^−1^). 10 μl of proteins in 25 mM phosphate buffer (pH 7.5), 150 mM NaF and at a concentration of 0.35 mg ml^−1^ were loaded in nanoDSF grade standard capillaries. Samples were measured in triplicates and the calculated melting temperatures averaged. Melting temperature were calculated by the software PR.ThermControl.

### Yeast library preparation

Query strains were constructed on the basis of an automated mating compatible strain. The query strains contained a peroxisomal marker (Pex3p-mCherry::HIS), a deletion of *PEX5* (*pex5*Δ::NAT) and a plasmid expressing WT or mutant PEX5 (PEX5^WT^, PEX5^2D^ or PEX5^2A^). Using an automated mating method (Cohen and Schuldiner 2011; Tong and Boone 2006), the query strains were crossed with a *NOP1*pr-*N*′-GFP-peroxi library (Dahan et al. 2017). The *NOP1*pr-*N*′-GFP-peroxisomal library is a collection of ∼90 strains containing all the known peroxisomal proteins together with potential peroxisomal proteins and controls, genomically tagged with GFP at their *N*-terminus and expressed under the generic constitutive *SpNOP1* promoter. To perform the manipulations in high-density format we used a RoToR bench top colony arrayer (Singer Instruments). In short: mating was performed on rich medium plates, and selection for diploid cells was performed on SD(MSG)-HIS-URA plates containing Geneticin (200 μg ml^−1^) and Nourseothricin (200 μg ml^−1^). Sporulation was induced by transferring cells to nitrogen starvation media plates for 7 days. Haploid cells containing the desired mutations were selected by transferring cells to SD-URA-HIS-LYS-ARG-LEU plates containing Geneticin and Nourseothricin (same concentrations as above), alongside the toxic amino acid derivatives Canavanine and Thialysine (Sigma-Aldrich) to select against remaining diploids and select for spores with an α mating type. A similar method was used to generate control, *pex5Δ*, and *pex8Δ* strains expressing GFP-Mdh3p (Supplementary Figure 4D), in which the query strain contained a peroxisomal marker (Pex3p-mCherry::NAT) and URA3::GFP-Mdh3p. This query strain was crossed with three strains from the mini peroxisomal deletion library (Gabay-Maskit et al. 2020): a control (no deletion), *pex5Δ*, and *pex8Δ* strain, all of which contain KAN (Geneticin) resistance.

### Automated high-throughput fluorescence microscopy

The yeast collections were visualized using an automated microscopy setup as described previously (Breker et al. 2013). In short: cells were transferred from agar plates into 384-well polystyrene plates (Greiner) for growth in liquid media using the RoToR arrayer robot. Liquid cultures were grown in a LiCONiC incubator, overnight at 30 °C in SD-URA-HIS medium. A JANUS liquid handler (PerkinElmer) connected to the incubator was used to dilute the strains to an OD_600_ of ∼0.2 into plates containing SD medium (6.7 g l^−1^ yeast nitrogen base and 2% glucose) supplemented with complete amino acids. Plates were incubated at 30 °C for 4 h. The cultures in the plates were then transferred by the liquid handler into glass-bottom 384-well microscope plates (Matrical Bioscience) coated with Concanavalin A (Sigma-Aldrich). After 20 min, cells were washed three times with SD-Riboflavin complete medium to remove non-adherent cells and to obtain a cell monolayer. The plates were then transferred to the ScanR automated inverted fluorescent microscope system (Olympus) using a robotic swap arm (Hamilton). Images of cells in the 384-well plates were recorded in the same liquid as the washing step at 24 °C using a 60× air lens (NA 0.9) and with an ORCA-ER charge-coupled device camera (Hamamatsu). Images were acquired in two channels: GFP (excitation filter 490/20 nm, emission filter 535/50 nm) and mCherry (excitation filter 572/35 nm, emission filter 632/60 nm). All images were taken at a single focal plane. The same protocol was used to visualize fluorescence signals in GFP-Mdh3p-expressing control, *pex5Δ*, and *pex8Δ* strains (Supplementary Figure 4D).

### Fluorescence anisotropy

Fluorescently-labeled peptides corresponding to the carboxyl-terminal 9 amino acids of Lys1p, Cat2p, Pcs60p and Idp3p with a tyrosine (Y) added to the peptide amino-terminus for concentration measurements (Lys1p, FITC-YARVKRSSRL; Cat2p, FITC-YNENKRKAKL; Pcs60p, FITC-YKSSRNKSKL; Idp3p, FITC-YNEDKKGMCKL; GenScript) were solubilized in water and used in the assay at a final concentration of 10 nM. Fluorescence anisotropy measurements were performed in black 96-well plates (Greiner) with an Infinite M1000 plate reader (Salpietro et al. 2014) with excitation/detection at 470/530 nm. Experiments were performed in 50 mM HEPES pH 7.5, 150 mM NaCl. Serial dilutions of recombinant Pex5p^WT^ and Pex5p^2D^ ranging from 6.6 µM to 1.5 nM (for Lys1p, Cat2p and Pcs60p) or 38 µM to 160 nM (for Idp3p) were measured in triplicates for each experiment. Three independent experiments were performed and binding data were analyzed using Prism (GraphPad software, USA). Kinetic information was obtained by least square fitting of a binding-saturation model with one binding site.

### Homology model

*Saccharomyces* Pex5p TPR domain (residues 313–612) was modeled with https://swissmodel.expasy.org/ (Benkert et al. 2011; Bertoni et al. 2017; Bienert et al. 2017; Guex et al. 2009;Waterhouse et al. 2018) based on the apo form of the TPR domain of human PEX5 (PDB: 2c0m). The homology model had a Global Model Quality Estimation score of 0.68 and a QMEAN of −1.37. Residues serine 507 and 523 were manually phosphorylated in Coot (Emsley et al. 2010) and non-clashing rotamers were selected.

### Data availability

The mass spectrometry proteomics data have been deposited to the ProteomeXchange Consortium via the PRIDE (Perez-Riverol et al. 2019) partner repository with the dataset identifier PXD015676.

## Supplementary Material

Supplementary Material Details

Supplementary Material Details
