# Supplementary material for: Phosphorylation of the receptor protein Pex5p modulates import of proteins into peroxisomes
Source: Biol Chem. 2022 Sep 21;404(2-3):135–55. doi: 10.1515/hsz-2022-0168 (PMC9929924; doi:10.1515/hsz-2022-0168)
Supplement: Supplementary file 1 — Supplementary Material Details [file j_hsz-2022-0168_suppl_001.pdf]

Phosphorylation of the receptor protein Pex5p modulates import of proteins into peroxisomes

Sven Fischer, Jérôme Bürgi, Shiran Gabay-Maskit, Renate Maier, Thomas Mastalski, Eden Yifrach, Agnieszka Obarska-Kosinska, Markus Rudowitz, Ralf Erdmann, Harald W. Platta, Matthias Wilmanns, Maya Schuldiner, Einat Zalckvar, Silke Oeljeklaus, Friedel Drepper and Bettina Warscheid

Supplementary Figures and Tables

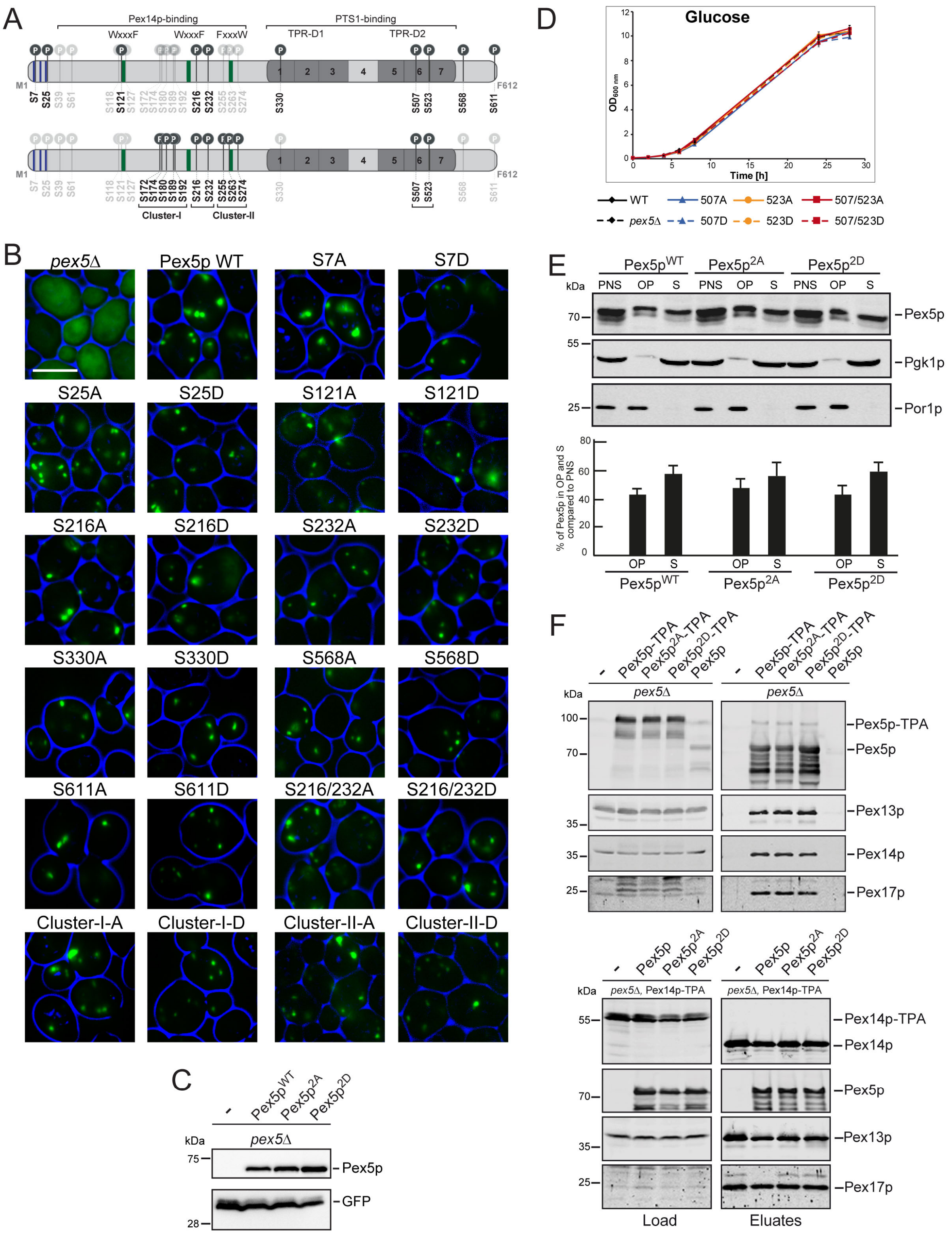

Supplementary Figure 1. Generation and analysis of Pex5p phosphosite mutants for effects on peroxisomal import and cell growth.

(A) Illustration of Pex5p phosphosite mutants generated in this work. The serine residues of ten individual sites (top), two dual sites (S216/232, S507/523) and two clusters of sites (bottom) were changed to alanine and aspartate. (B) Representative fluorescence microscopy images of yeast cells grown on oleate and expressing plasmid-encoded Pex5p wildtype ($Pex5p^{WT}$) or phosphosite mutants ($Pex5p^{2A}$, $Pex5p^{2D}$) as indicated and GFP-SKL. Blue, cell boundaries; scale bar, 5 μm. (C) Expression levels of $Pex5p^{WT}$, $Pex5p^{2A}$ and $Pex5p^{2D}$ as well as GFP-SKL in lysates of cells used for fluorescence microscopy (see Figure 1C) analyzed by immunoblotting using antibodies against Pex5p and GFP, respectively. Cells were lysed with TCA. (D) Growth of yeast cells expressing $Pex5p^{WT}$ or phosphosite mutants as indicated in SC medium containing 2% glucose. Same strains as used for the fluorescence microscopy experiment shown in Figure 1C. Error bars indicate standard deviation (n = 3). (E) Membrane binding is not affected in the phosphomimetic $Pex5p^{2D}$ mutant. Postnuclear supernatants (PNS) prepared from oleate-induced cells expressing $Pex5p^{WT}$, $Pex5p^{2A}$ or $Pex5p^{2D}$ were separated by centrifugation into an organellar pellet (OP) and a cytosolic supernatant (S). Top, Equal amounts of PNS, OP and S were analyzed by immunoblotting using antibodies recognizing Pex5p, the cytosolic protein 3-phosphoglycerate kinase (Pgk1p) and the membrane protein porin (Por1p). Bottom, Quantification of immunoblot signals for Pex5p in indicated fractions. Shown are the mean of Pex5p signals observed in OP and S compared to the corresponding PNS. Error bars indicate standard deviation (n = 3). (F) Association of Pex5p with the docking complex remains unaltered in Pex5p-S507/523 double phosphosite mutants. Top, TPA-tagged Pex5p versions (Pex5p wildtype, $Pex5p^{2A}$ or $Pex5p^{2D}$) were affinity-purified from digitonin-solubilized membrane fractions. Cells devoid of Pex5p (-) or untagged Pex5p served as control. Proteins of the solubilized membrane fractions (load) and eluate fractions were separated by SDS-PAGE followed by immunoblotting using antibodies against components of the peroxisomal docking complex as indicated. Bottom, Affinity purification of TPA-tagged Pex14p from digitonin-solublized membrane fractions of cells expressing Pex5p, $Pex5p^{2A}$ or $Pex5p^{2D}$.

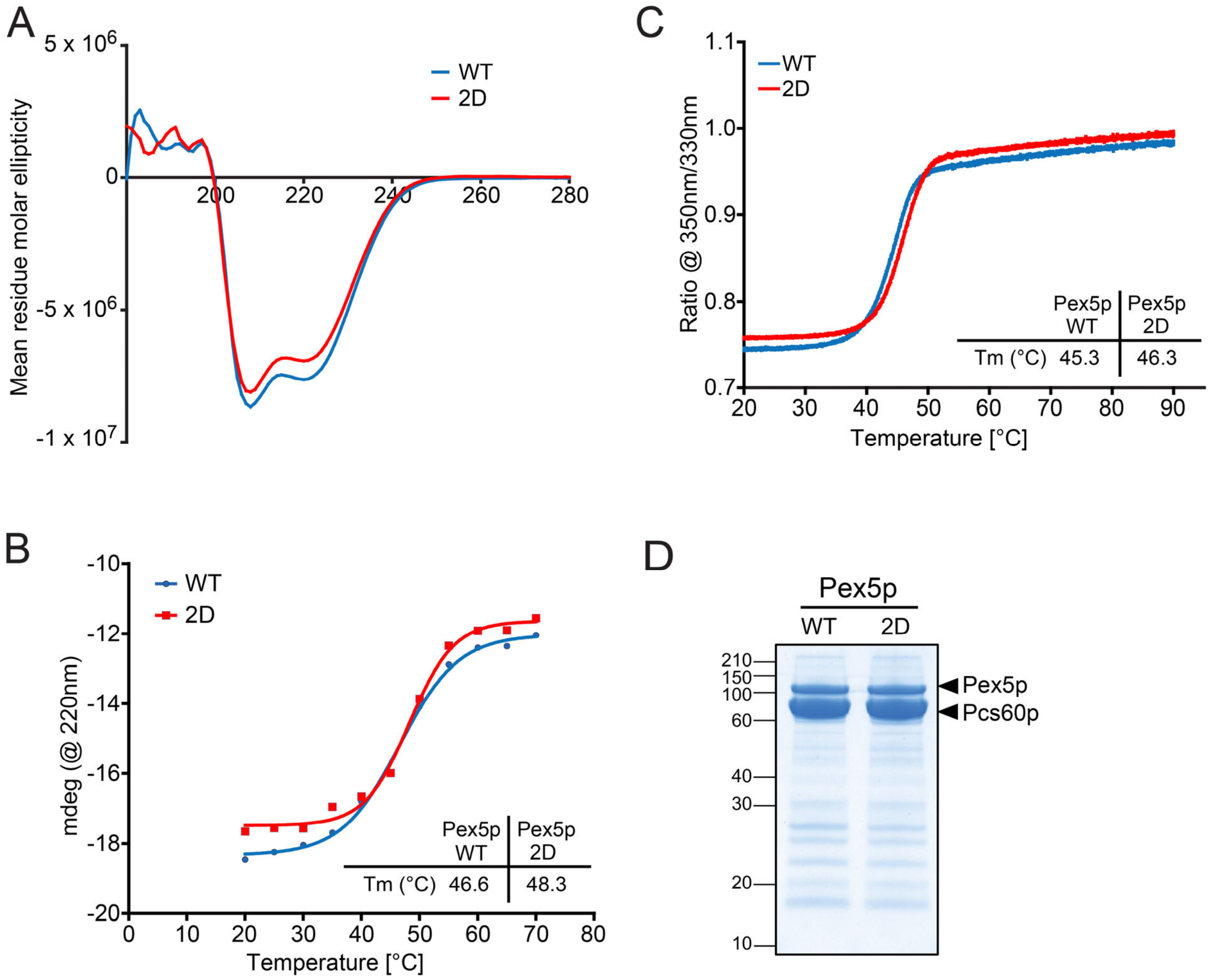

Supplementary Figure 2. Analysis of overall protein structure of Pex5p^{2D} and Pex5p^{WT}.

(A) Far-UV CD spectra of Pex5p^{WT} and the phospho-mimicking Pex5p^{2D} mutant. The mean residue molar ellipticity is given for a range between 180 and 280 nm. Measurements were performed with a protein concentration of 0.2 mg/ml for each Pex5p variant. (B) Melting curve of far-UV CD spectra of Pex5p^{WT} and Pex5p^{2D} monitored at 220 nm. Melting temperatures were calculated by fitting data from triplicate measurements to a sigmoidal Boltzmann function. (C) Melting curve of Pex5p^{WT} and Pex5p^{2D} monitored by nano differential scanning fluorimetry (nanoDSF) applying thermal ramping from 20 - 90°C and using a protein concentration of 0.35 mg/ml for each Pex5p variant. (D) SDS-PAGE analysis and Commassie staining of protein samples after ITC measurements (see Figure 2A).

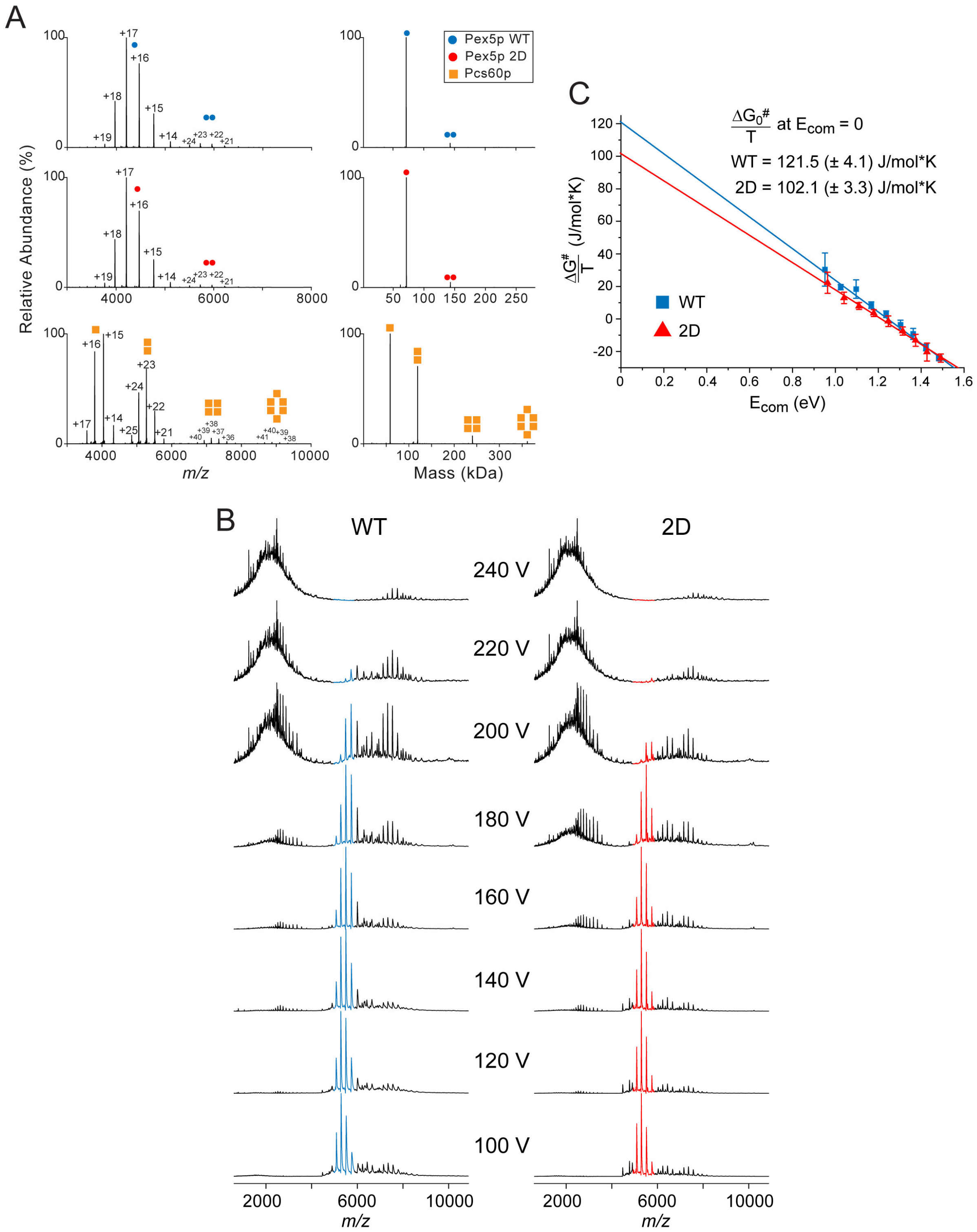

Supplementary Figure 3. Native MS measurements of Pex5p variants and Pcs60p and linear relation between apparent Gibbs free energies of dissociation and gas-phase collision energies for receptor-cargo complexes.

(A) Native MS measurements of recombinant Pex5p^{WT}, Pex5p^{2D} (each 7.5 µM) and Pcs60p (5 µM), providing information on their oligomerization states, charge states and molecular masses. Left, native mass spectra; right, deconvoluted zero-charge mass spectra. Masses of Pex5p monomeric and dimeric forms were assigned as follows: 71.459 kDa and 142.918 kDa for Pex5p^{WT}, and 71.515 kDa and 143.03 kDa for Pex5p^{2D}. Masses for Pcs60p mono-, di-, tetra- and hexamers are 60.63 kDa, 121.26 kDa, 242.53 kDa and 363.79 kDa, respectively. *m/z*, mass-to-charge ratio. (B) Native MS analysis of reconstituted Pex5p-Pcs60p complexes with Pex5p^{WT} or Pex5p^{2D} mutant (each 7.5 µM) together with Pcs60p (5 µM). Measurements were performed in dependency of the acceleration voltage applied for dissecting differences in complex stability. Signals from in solution-derived monomers were filtered out using a quadrupole mass filter profile fixed at *m/z* 5,800 to impose a low mass cut-off below *m/z* ~4,600. Collision energy was gradually increased from 100 to 240 V in 10-V steps to study complex stability; representative native MS spectra between 100 and 240 V are shown. Charge states +23 to +26 of 1:1 receptor-cargo complexes with Pex5p^{WT} (blue) and Pex5p^{2D} (red) were quantified. (C) Apparent Gibbs free energy plotted against corresponding E_{com} values for receptor-cargo complexes with Pex5p^{WT} or Pex5p^{2D}. Linear extrapolation using observed normalized amplitudes of 1:1 receptor-cargo complexes in the range between 130 and 210 V acceleration voltage yields apparent activation energies for gas phase complex dissociation at E_{com} = 0 representing no external activation (n = 3).

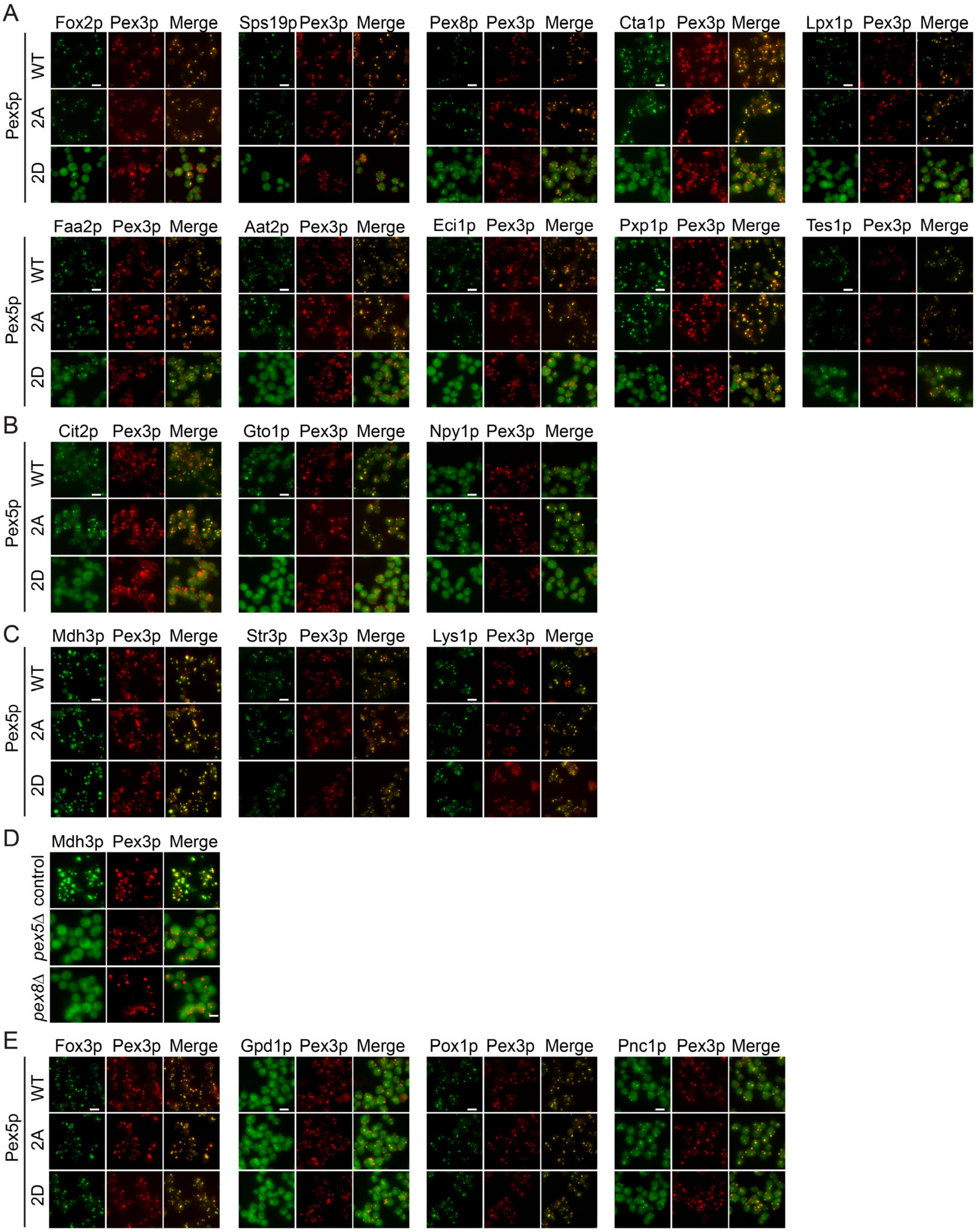

Supplementary Figure 4. Subcellular distribution of GFP-tagged peroxisomal proteins in cells expressing Pex5p^{WT}, Pex5p^{2D} or Pex5p^{2A} and cells lacking the *PEX5* or *PEX8* gene.

(A-C, E) Same experiment as described in Figure 4. Shown are representative images of peroxisomal GFP-tagged PTS1 proteins exhibiting a moderate import phenotype (A; category 1), a strong import phenotype (B; category 2) or no import phenotype (C; category 3) and proteins imported into peroxisomes in a Pex5p-independent manner (E; category 'others') as listed in Figure 4A (except for those depicted in Figures 4B - 4E). (D) Fluoresence microscopy analysis of the subcellular distribution of GFP-Mdh3p in control, *pex5*Δ and *pex8*Δ cells. (A-E) Pex3p, mCherry-tagged Pex3p, a peroxisomal marker protein; scale bars, 5 µm.

Supplementary Table 1. Pex5p (phospho)peptides identified by LC-MS in affinity-purified native Pex5p of *Saccharomyces cerevisiae*. [see separate Excel file]

Supplementary Table 2. Information about plasmids used and generated in this study as well as primers, restriction enzymes, DNA templates and target plasmids.

Plasmid	Characteristics/Insert	Backbone	Source or Reference	Primers	Restriction Enzyme	Template	Target Plasmid
pRS416	CEN6, ARSH4, URA3, bla, lacZ	pBlue-script II	Sikorski and Hieter 1989				
pUG6	loxP-kanMX-loxP, bla	pFA6-kanMX4					
pUG27	loxP-Ag*TEF1*$_{Pro}$-Sp*HIS5*-Ag*TEF1*-$_{Term}$-loxP, bla	pUG6	Güldener et al. 2002				
pUG73	loxP-Kl*LEU2*$_{Pro}$-Kl*LEU2*-Ki*LEU2*$_{Term}$-loxP, bla	pUG6	Güldener et al. 2002				
p232	PEX5$_{Pro}$-PEX5-ADH1$_{Term}$	pRS416	Kerssen et al. 2006				
pJF321	PEX5$_{Pro}$-PEX5^{S7A}-ADH1$_{Term}$	pRS416	this study	O601, O602		p232	p232
pFS519	PEX5$_{Pro}$-PEX5^{S7D}-ADH1$_{Term}$	pRS416	this study	O1112, O604		p232	p232
pJF337	PEX5$_{Pro}$-PEX5S^{25A}-ADH1$_{Term}$	pRS416	this study	O605, O606		p232	p232
pJF338	PEX5$_{Pro}$-PEX5S^{25D}-ADH1$_{Term}$	pRS416	this study	O607, O608		p232	p232
pRI373	PEX5$_{Pro}$-PEX5^{S121A}-ADH1$_{Term}$	pRS416	this study	O764, O765		p232	p232
pRI374	PEX5$_{Pro}$-PEX5^{S121D}-ADH1$_{Term}$	pRS416	this study	O766, O767		p232	p232
pRI375	PEX5$_{Pro}$-PEX5$^{S174/180/189/192A}$-ADH1$_{Term}$	pRS416	this study	O461, O462	NheI, Bsu36I	GeneArt string 1*	p232
pRI376	PEX5$_{Pro}$-PEX5$^{S174/180/189/192D}$-ADH1$_{Term}$	pRS416	this study	O461, O462	NheI, Bsu36I	GeneArt string 2*	p232
pFS515	PEX5$_{Pro}$-PEX5$^{S172/174/180/189/192A}$-ADH1$_{Term}$	pRS416	this study	O1113, O1114		pRI375	pRI375
pFS516	PEX5$_{Pro}$-PEX5S$^{172/174/180/189/192D}$-ADH1$_{Term}$	pRS416	this study	O1115, O1116		pRI376	pRI376
pSF247	PEX5$_{Pro}$-PEX5^{S216A}-ADH1$_{Term}$	pRS416	this study	O451, O452		p232	p232
pSF258	PEX5$_{Pro}$-PEX5^{S216D}-ADH1$_{Term}$	pRS416	this study	O453, O454		p232	p232
pSF246	PEX5$_{Pro}$-PEX5^{S232A}-ADH1$_{Term}$	pRS416	this study	O396, O397		p232	p232
pSF257	PEX5$_{Pro}$-PEX5^{S232D}-ADH1$_{Term}$	pRS416	this study	O398, O399		p232	p232
pSF259	PEX5$_{Pro}$-PEX5$^{S216/232A}$-ADH1$_{Term}$	pRS416	this study	O396, O397		pSF247	pSF247
pSF260	PEX5$_{Pro}$-PEX5$^{S216/232D}$-ADH1$_{Term}$	pRS416	this study	O453, O454		pSF257	pSF257
pRI377	PEX5$_{Pro}$-PEX5$^{S255/263/274A}$-ADH1$_{Term}$	pRS416	this study	O461, O462	NheI, Bsu36I	GeneArt string 3*	p232

Plasmid	Characteristics/Insert	Backbone	Source or Reference	Primers	Restriction Enzyme	Template	Target Plasmid
pRI378	$PEX5_{Pro}$-$PEX5^{S255/263/274D}$-$ADH1_{Term}$	pRS416	this study	O461, O462	NheI, Bsu36I	GeneArt string 4*	p232
pSF513	$PEX5_{Pro}$-$PEX5^{S330A}$-$ADH1_{Term}$	pRS416	this study	O1054, O1055		p232	p232
pSF514	$PEX5_{Pro}$-$PEX5^{S330D}$-$ADH1_{Term}$	pRS416	this study	O1056, O1057		p232	p232
pSF401	$PEX5_{Pro}$-$PEX5^{S507A}$-$ADH1_{Term}$	pRS416	this study	O843, O844		p232	p232
pSF402	$PEX5_{Pro}$-$PEX5^{S507D}$-$ADH1_{Term}$	pRS416	this study	O845, O846		p232	p232
pSF403	$PEX5_{Pro}$-$PEX5^{S523A}$-$ADH1_{Term}$	pRS416	this study	O847, O848		p232	p232
pSF404	$PEX5_{Pro}$-$PEX5^{S523D}$-$ADH1_{Term}$	pRS416	this study	O849, O850		p232	p232
pSF405	$PEX5_{Pro}$-$PEX5^{S568A}$-$ADH1_{Term}$	pRS416	this study	O851, O852		p232	p232
pSF406	$PEX5_{Pro}$-$PEX5^{S568D}$-$ADH1_{Term}$	pRS416	this study	O853, O854		p232	p232
pSF407	$PEX5_{Pro}$-$PEX5^{S611A}$-$ADH1_{Term}$	pRS416	this study	O855, O856		p232	p232
pSF408	$PEX5_{Pro}$-$PEX5^{S611D}$-$ADH1_{Term}$	pRS416	this study	O857, O858		p232	p232
pRI379	$PEX5_{Pro}$-$PEX5^{S507/523A}$-$ADH1_{Term}$	pRS416	this study	O718, O719	MscI, BglII	GeneArt string 5*	p232
pRI380	$PEX5_{Pro}$-$PEX5^{S507/523D}$-$ADH1_{Term}$	pRS416	this study	O718, O719	MscI, BglII	GeneArt string 6*	p232
pGFP-SKL	$Met25_{Pro}$-GFP-SKL-$Cyc1_{Term}$	pRS414	Schafer et al. 2004				
pSF432	$PEX5_{Pro}$-PEX5-$ADH1_{Term}$; URA3::KanMX	pRS416	this study	O912, O913, O914, O915	Gibsen et al. 2009	p232, pUG6	Gibson et al. 2009
pSF433	$PEX5_{Pro}$-$PEX5^{S507/523A}$-$ADH1_{Term}$; URA3::KanMX	pRS416	this study	O912, O913, O914, O915		p232, pUG6	Gibson et al. 2009
pSF434	$PEX5_{Pro}$-$PEX5^{S507/523D}$-$ADH1_{Term}$; URA3::KanMX	pRS416	this study	O912, O913, O914, O915		p232, pUG6	Gibson et al. 2009
pSH462	$T7_{Pro}$-6His-PEX5-$T7_{Term}$	pET9d	Kerssen et al. 2006				
pSF480	$T7_{Pro}$-6His-$PEX5^{S507D}$-$T7_{Term}$	pET9d	this study	O845, O846		pSH462	pSH462
pSF482	$T7_{Pro}$-6His-$PEX5^{S507/523D}$-$T7_{Term}$	pET9d	this study	O849, O850		pSF480	pSF480
pSH490	Tac_{Pro}-GST-PCS60	pGEX-4T3	Hagen et al. 2015				

Pro, promoter; Term, terminator; *, see Supplementary Table 4.

Supplementary Table 3. Sequences of the primers used in this study.

ID	Name	Sequence [5' to 3']
O394	Pex5_KO_fwd	TTTTTGGATATATATACATCAATAAACAATATATCATAACACATGCAGCTGAAGCTTCGTACGC
O395	Pex5_KO_rev	TTTGGGCAGTGATGCGAGAACATAAAATTGCGGAGAACCATATCAGCATAGGCCACTAGTGGATCTG
O396	Pex5_S232A_fwd	GGAGGAAAATGTGGCTGAAGTAGAACAAAACAAACC
O397	Pex5_S232A_rev	GGTTTGTTTTGTTCTACTTCAGCCACATTTTCCTCC
O398	Pex5_S232D_fwd	GGAGGAAAATGTGGATGAAGTAGAACAAAACAAACC
O399	Pex5_S232D_rev	GGTTTGTTTTGTTCTACTTCATCCACATTTTCCTCC
O451	Pex5_S216A_fwd2	GCTGGAAAAAGAAGTCGCAGAAAACTTGGAC
O452	Pex5_S216A_rev2	GTCCAAGTTTTCTGCGACTTCTTTTTCCAGC
O453	Pex5_S216D_fwd2	GCTGGAAAAAGAAGTCGACGAAAACTTGGAC
O454	Pex5_S216D_rev2	GTCCAAGTTTTCGTCGACTTCTTTTTCCAGC
O461	GeneArt_Pex5_232_fwd	GCATCATCGATGATAAAAGAAGAATGG
O462	GeneArt_Pex5_232_rev	GTACTGCTAGCGCTGCCTCGC
O601	Pex5_S7A_fwd	CGTAGGAAGTTGCGCAGTGGG
O602	Pex5_S7A_rev	CCCACTGCGCAACTTCCTACG
O604	Pex5_S7D_rev	CCCACGTCGCAACTTCCTACG
O605	Pex5_S25A_fwd	GCAGAACAAAGCGCTTCAG
O606	Pex5_S25A_rev	CTGAAGCGCTTTGTTCTGC
O607	Pex5_S25D_fwd	GCAGAACAAAGACCTTCAG
O608	Pex5_S25D_rev	CTGAAGGTCTTTGTTCTGC
O718	GeneArt_Pex5_507_fwd	GCATCTGGCCAATTCCAATAGAGATGAGGA
O719	GeneArt_Pex5_507_rev	GTACTAGATCTTCAAAACGAAAATTCTCCTTTAAATCTT
O764	Pex5_S121A_fwd	CGATATATCTCATTGGGCACAGG
O765	Pex5_S121A_rev	CCTGTGCCCAATGAGATATATCG
O766	Pex5_S121D_fwd	CGATATATCTCATTGGGATCAGG
O767	Pex5_S121D_rev	CCTGATCCCAATGAGATATATCG
O843	Pex5_S507A_fwd	CCAATTCCAATAGAGCAGAGGAAGC
O844	Pex5_S507A_rev	GCTTCCTCTGCTCTATTGGAATTGG
O845	Pex5_S507D_fwd	CCAATTCCAATAGAGACGAGGAAGC
O846	Pex5_S507D_rev	GCTTCCTCGTCTCTATTGGAATTGG

ID	Name	Sequence [5' to 3']
O847	Pex5_S523A_fwd	CTAAAACCTGCTTTTGTTAGAGC
O848	Pex5_S523A_rev	GCTCTAACAAAAGCAGGTTTTAG
O849	Pex5_S523D_fwd	CTAAAACCTGATTTTGTTAGAGC
O850	Pex5_S523D_rev	GCTCTAACAAAATCAGGTTTTAG
O851	Pex5_S568A_fwd	CGTTGGAGCTCTCTTGAATACG
O852	Pex5_S568A_rev	CGTATTCAAGAGAGCTCCAACG
O853	Pex5_S568D_fwd	CGTTGGAGATCTCTTGAATACG
O854	Pex5_S568D_rev	CGTATTCAAGAGATCTCCAACG
O855	Pex5_S611A_fwd	GGAGAATTTGCGTTTTGAAGATCTAC
O856	Pex5_S611A_rev	GTAGATCTTCAAAACGCAAATTCTCC
O857	Pex5_S611D_fwd	GGAGAATTTGACTTTTGAAGATCTAC
O858	Pex5_S611D_rev	GTAGATCTTCAAAAGTCAAATTCTCC
O912	P232_URA_to_Kan_1_fwd	CTGGTCGCTATACTGCTGTATTATAAGTAAATGCATGTATACTAAACTCAC
O913	P232_URA_to_Kan_2_rev	CTGGGCCTCCATGTCTTGAAAAGCTGTGGTATGGTGC
O914	P232_URA_to_Kan_3_fwd	ACCACAGCTTTTCAAGACATGGAGGCCCAGAATACC
O915	P232_URA_to_Kan_4_rev	TTACTTATAATACAGCAGTATAGCGACCAGCATTCACA
O1054	Pex5_S330A_fwd	GCCAAATTGGCCGAGGCAG
O1055	Pex5_S330A_rev	CTGCCTCGGCCAATTTGGC
O1056	Pex5_S330D_fwd	GCCAAATTGGACGAGGCAG
O1057	Pex5_S330D_rev	CTGCCTCGTCCAATTTGGC
O1112	S7D_for_ pJF321_fwd	GTTGCGACGTGGGAAATAATCC
O1113	S172A_for_pRI375_fwd	GAACGCTCTTGCTGGATCAAGG
O1114	S172A_for_pRI375_rev	CAGCAAGAGCGTTCATAGAAGC
O1115	S172D_for_pRI376_fwd	GAACGATCTTGATGGATCAAGGC
O1116	S172D_for_pRI376_rev	CATCAAGATCGTTCATAGAAGCATAAG

Supplementary Table 4. Sequences of GeneArt constructs used for the simultaneous introduction of multiple phosphosite mutations in *PEX5*.

Name	Sequence (5' to 3')	Primers	Restriction Enzymes
GeneArt string 1	GCATCATCGATGATAAAAGAAGAATGGAAATAGGGCCATCCTCAGGCAGGCTTCCAC<br>CTTTTTCAAACGTACATTCTCTACAGACTTCAGCAAACCCAACCCAAATTAAGGGAGTG<br>AACGATATATCTCATTGGTCACAGGAATTTCAAGGTAGTAATAGTATTCAAAATAGAA<br>ACGCGGATACAGGAAATTCAGAAAAGGCATGGCAGCGTGGCTCAACAACCGCATCAA<br>GCCGGTTTCAGTACCCTAATACCATGATGAATAACTATGCTTATGCTTCTATGAACAGT<br>CTTGCTGGATCAAGGCTCCAAGCGCCTGCTTTCATGAATCAACAACAGGCTGGTCGTG<br>CTAAAGAAGGAGTCAATGAGCAAGAACAACAACCCTGGACAGATCAGTTTGAAAAGC<br>TGGAAAAAGAAGTCTCAGAAAACTTGGACATAAATGATGAAATAGAGAAGGAGGAA<br>AATGTGAGTGAAGTAGAACAAAACAAACCAGAAACTGTTGAGAAGGAAGAAGGAGT<br>ATATGGAGATCAGTATCAATCTGATTTCCAAGAAGTGTGGGATAGCATACACAAGGA<br>CGCTGAAGAAGTCTTGCCATCCGAATTAGTTAATGATGACCTCAATCTAGGAGAAGAC<br>TACTTGAAATATCTCGGCGGTAGAGTAAATGGGAACATCGAGTATGCTTTTCAATCTA<br>ACAACGAATATTTTAATAATCCTAATGCTTATAAAATTGGCTGCCTACTGATGGAAAAC<br>GGAGCCAAATTGAGCGAGGCAGCGCTAGCAGTAC	O461, O462	NheI, Bsu36I
GeneArt string 2	GCATCATCGATGATAAAAGAAGAATGGAAATAGGGCCATCCTCAGGCAGGCTTCCAC<br>CTTTTTCAAACGTACATTCTCTACAGACTTCAGCAAACCCAACCCAAATTAAGGGAGTG<br>AACGATATATCTCATTGGTCACAGGAATTTCAAGGTAGTAATAGTATTCAAAATAGAA<br>ACGCGGATACAGGAAATTCAGAAAAGGCATGGCAGCGTGGCTCAACAACCGCATCAA<br>GCCGGTTTCAGTACCCTAATACCATGATGAATAACTATGCTTATGCTTCTATGAACAGT<br>CTTGATGGATCAAGGCTCCAAGACCCTGCTTTCATGAATCAACAACAGGATGGTCGTG<br>ATAAAGAAGGAGTCAATGAGCAAGAACAACAACCCTGGACAGATCAGTTTGAAAAGC<br>TGGAAAAAGAAGTCTCAGAAAACTTGGACATAAATGATGAAATAGAGAAGGAGGAA<br>AATGTGAGTGAAGTAGAACAAAACAAACCAGAAACTGTTGAGAAGGAAGAAGGAGT<br>ATATGGAGATCAGTATCAATCTGATTTCCAAGAAGTGTGGGATAGCATACACAAGGA<br>CGCTGAAGAAGTCTTGCCATCCGAATTAGTTAATGATGACCTCAATCTAGGAGAAGAC<br>TACTTGAAATATCTCGGCGGTAGAGTAAATGGGAACATCGAGTATGCTTTTCAATCTA<br>ACAACGAATATTTTAATAATCCTAATGCTTATAAAATTGGCTGCCTACTGATGGAAAAC<br>GGAGCCAAATTGAGCGAGGCAGCGCTAGCAGTA	O461, O462	NheI, Bsu36I
GeneArt string 3	GCATCATCGATGATAAAAGAAGAATGGAAATAGGGCCATCCTCAGGCAGGCTTCCAC<br>CTTTTTCAAACGTACATTCTCTACAGACTTCAGCAAACCCAACCCAAATTAAGGGAGTG<br>AACGATATATCTCATTGGTCACAGGAATTTCAAGGTAGTAATAGTATTCAAAATAGAA<br>ACGCGGATACAGGAAATTCAGAAAAGGCATGGCAGCGTGGCTCAACAACCGCATCAA<br>GCCGGTTTCAGTACCCTAATACCATGATGAATAACTATGCTTATGCTTCTATGAACAGT<br>CTTAGTGGATCAAGGCTCCAATCGCCTGCTTTCATGAATCAACAACAGTCTGGTCGTTC<br>TAAAGAAGGAGTCAATGAGCAAGAACAACAACCCTGGACAGATCAGTTTGAAAAGCT<br>GGAAAAAGAAGTCTCAGAAAACTTGGACATAAATGATGAAATAGAGAAGGAGGAAA<br>ATGTGAGTGAAGTAGAACAAAACAAACCAGAAACTGTTGAGAAGGAAGAAGGAGTA<br>TATGGAGATCAGTATCAAGCTGATTTCCAAGAAGTGTGGGATGCCATACACAAGGAC<br>GCTGAAGAAGTCTTGCCAGCCGAATTAGTTAATGATGACCTCAATCTAGGAGAAGACT<br>ACTTGAAATATCTCGGCGGTAGAGTAAATGGGAACATCGAGTATGCTTTTCAATCTAA<br>CAACGAATATTTTAATAATCCTAATGCTTATAAAATTGGCTGCCTACTGATGGAAAACG<br>GAGCCAAATTGAGCGAGGCAGCGCTAGCAGTAC	O461, O462	NheI, Bsu36I

Name	Sequence (5' to 3')	Primers	Restriction Enzymes
GeneArt string 4	GCATCATCGATGATAAAAGAAGAATGGAAATAGGGCCATCCTCAGGCAGGCTTCCAC<br>CTTTTTCAAACGTACATTCTCTACAGACTTCAGCAAACCCAACCCAAATTAAGGGAGTG<br>AACGATATATCTCATTGGTCACAGGAATTTCAAGGTAGTAATAGTATTCAAAATAGAA<br>ACGCGGATACAGGAAATTCAGAAAAGGCATGGCAGCGTGGCTCAACAACCGCATCAA<br>GCCGGTTTCAGTACCCTAATACCATGATGAATAACTATGCTTATGCTTCTATGAACAGT<br>CTTAGTGGATCAAGGCTCCAATCGCCTGCTTTCATGAATCAACAACAGTCTGGTCGTTC<br>TAAAGAAGGAGTCAATGAGCAAGAACAACAACCCTGGACAGATCAGTTTGAAAAGCT<br>GGAAAAAGAAGTCTCAGAAAACTTGGACATAAATGATGAAATAGAGAAGGAGGAAA<br>ATGTGAGTGAAGTAGAACAAAACAAACCAGAAACTGTTGAGAAGGAAGAAGGAGTA<br>TATGGAGATCAGTATCAAGATGATTTCCAAGAAGTGTGGGATGACATACACAAGGAC<br>GCTGAAGAAGTCTTGCCAGACGAATTAGTTAATGATGACCTCAATCTAGGAGAAGACT<br>ACTTGAAATATCTCGGCGGTAGAGTAAATGGGAACATCGAGTATGCTTTTCAATCTAA<br>CAACGAATATTTTAATAATCCTAATGCTTATAAAATTGGCTGCCTACTGATGGAAAACG<br>GAGCCAAATTGAGCGAGGCAGCGCTAGCAGTAC	O461, O462	NheI, Bsu36I
GeneArt string 5	GCATCTGGCCAATTCCAATAGAGCAGAGGAAGCAATCCAAGCCTATCATAGGGCACT<br>ACAACTAAAACCTGCTTTTGTTAGAGCTCGCTATAATCTGGCGGTATCATCCATGAATA<br>TAGGCTGTTTCAAAGAAGCAGCAGGCTACTTATTAAGTGTTCTAAGTATGCATGAAGT<br>GAACACTAATAATAAAAAAGGAGACGTTGGATCTCTCTTGAATACGTACAATGATACT<br>GTTATAGAGACTTTGAAGAGAGTTTTTATAGCGATGAATAGAGATGATTTACTTCAAG<br>AAGTGAAGCCAGGCATGGACCTGAAAAGATTTAAAGGAGAATTTTCGTTTTGAAGAT<br>CTAGTAC	O718, O719	MscI, BglII
GeneArt string 6	GCATCTGGCCAATTCCAATAGAGATGAGGAAGCAATCCAAGCCTATCATAGGGCACTA<br>CAACTAAAACCTGATTTTGTTAGAGCTCGCTATAATCTGGCGGTATCATCCATGAATAT<br>AGGCTGTTTCAAAGAAGCAGCAGGCTACTTATTAAGTGTTCTAAGTATGCATGAAGTG<br>AACACTAATAATAAAAAAGGAGACGTTGGATCTCTCTTGAATACGTACAATGATACTG<br>TTATAGAGACTTTGAAGAGAGTTTTTATAGCGATGAATAGAGATGATTTACTTCAAGA<br>AGTGAAGCCAGGCATGGACCTGAAAAGATTTAAAGGAGAATTTTCGTTTTGAAGATCT<br>AGTAC	O718, O719	MscI, BglII

Supplementary Table 5. Information about *S. cerevisiae* strains used in this study.

ID	Name	Genotype	Source or Reference	Primers for HR
SC01	CB80	MATa, *ura3-52*, *leu2-1*, *trp1-63*, *his3-200*	Brocard et al. 1997	
SC38	CB80 ΔlysΔarg	CB80, *arg4*::loxP, *lys1*::loxP	this study	
SC245	SC38Δ*pex5*	CB80, *arg4*::loxP, *lys1*::loxP, *pex5*::kanMX4	this study	O394, O395 on pUG6
SC166	UTL7A-PEX5^{TPA}	MATa, *ura3-52*, *leu2-3/112*, *trp1*; *PEX5*:TPA-kanMX4	Schafer et al. 2004	
SC28	UTL7A-PEX14^{TPA}	MATa, *ura3-52*, *leu2-3/112*, *trp1*; *PEX14*:TPA-kanMX4	Agne et al. 2003	
SC368	Query strain Δ*pex5*	MATalpha, *his3*Δ1, *leu2*Δ0, *lys2*Δ0, *ura3*Δ0, *met15*Δ0, *lys2+/lys*, *can1*Δ::STE2pr-spHIS5, *lyp1*Δ::STE3pr-LEU2, PEX3-mCherry::HIS, *pex5*Δ::NAT	this study	
	SWAT-GFP library	MATa, *his3*Δ1; *leu2*Δ0; *met15*Δ0; *ura3*Δ0; *hph*Δn::URA3::SpNOP1pr-sfGFP-XXX	Yofe et al. 2016; Weill et al 2018	
yMS4585	Query strain Pex3-mCherry +GFP-Mdh3	MATalpha, *his3*Δ1, *leu2*Δ0, *lys2*Δ0, *ura3*Δ0, *met15*Δ0, *lys2+/lys*, *can1*Δ::STE2pr-spHIS5, *lyp1*Δ::STE3pr-LEU2, PEX3-mCherry::NAT, URA-GFP-MDH3	This study	
	Δpex5 from mini Peroxi-deletion library	MATa, *his3*Δ1; *leu2*Δ0; *met15*Δ0; *ura3*Δ0; *Δpex5::KAN*	Gabay-Maskit et al. 2020	
	Δpex8 from mini Peroxi-deletion library	MATa, *his3*Δ1; *leu2*Δ0; *met15*Δ0; *ura3*Δ0; *Δpex8::KAN*	Gabay-Maskit et al. 2020	
yMS116	Control strain from mini Peroxi-deletion library	MATa, *his3*Δ1; *leu2*Δ0; *met15*Δ0; *ura3*Δ0::KAN	Gabay-Maskit et al. 2020	

HR, homologous recombination; TPA, sequence coding for a cleavage site for the tobacco etch virus protease and Protein A; XXX, genes coding for peroxisomal proteins analyzed in the fluorescence microscopy screen (see Figure 4 and Supplementary Figure 4)

Supplementary references

Agne, B., Meindl, N.M., Niederhoff, K., Einwächter, H., Rehling, P., Sickmann, A., Meyer, H.E., Girzalsky, W., and Kunau, W.-H. (2003). Pex8p. An intraperoxisomal organizer of the peroxisomal import machinery. Mol. Cell 11: 635-646.

Brocard, C., Lametschwandtner, G., Koudelka, R., and Hartig, A. (1997). Pex14p is a member of the protein linkage map of Pex5p. EMBO J. 16: 5491-5500.

Gabay-Maskit, S., Cruz-Zaragoza, L.D., Shai, N., Eisenstein, M., Bibi, C., Cohen, N., Hansen, T., Yifrach, E., Harpaz, N., Belostotsky, R., et al. (2020). A piggybacking mechanism enables peroxisomal localization of the glyoxylate cycle enzyme Mdh2 in yeast. J. Cell Sci. 133: jcs244376.

Gibson, D.G., Young, L., Chuang, R.Y., Venter, J.C., Hutchison, C.A., 3rd, and Smith, H.O. (2009). Enzymatic assembly of DNA molecules up to several hundred kilobases. Nat. Methods 6: 343-345.

Guldener, U., Heck, S., Fiedler, T., Beinhauer, J., and Hegemann, J.H. (1996). A new efficient gene disruption cassette for repeated use in budding yeast. Nucleic Acids Res. 24: 2519-2524.

Guldener, U., Heinisch, J., Köhler, G.J., Voss, D., and Hegemann, J.H. (2002). A second set of loxP marker cassettes for Cre-mediated multiple gene knockouts in budding yeast. Nucleic Acids Res. 30: e23.

Hagen, S., Drepper, F., Fischer, S., Fodor, K., Passon, D., Platta, H.W., Zenn, M., Schliebs, W., Girzalsky, W., Wilmanns, M., et al. (2015). Structural insights into cargo recognition by the yeast PTS1 receptor. J. Biol. Chem. 290: 26610-26626.

Kerssen, D., Hambruch, E., Klaas, W., Platta, H.W., de Kruijff, B., Erdmann, R., Kunau, W.H., and Schliebs, W. (2006). Membrane association of the cycling peroxisome import receptor Pex5p. J. Biol. Chem. 281: 27003-27015.

Schafer, A., Kerssen, D., Veenhuis, M., Kunau, W.H., and Schliebs, W. (2004). Functional similarity between the peroxisomal PTS2 receptor binding protein Pex18p and the N-terminal half of the PTS1 receptor Pex5p. Mol. Cell. Biol. 24: 8895-8906.

Sikorski, R.S., and Hieter, P. (1989). A system of shuttle vectors and yeast host strains designed for efficient manipulation of DNA in Saccharomyces cerevisiae. Genetics 122: 19-27.

Weill, U., Yofe, I., Sass, E., Stynen, B., Davidi, D., Natarajan, J., Ben-Menachem, R., Avihou, Z., Goldman, O., Harpaz, N., et al. (2018). Genome-wide SWAp-Tag yeast libraries for proteome exploration. Nat. Methods 15: 617-622.

Yofe, I., Weill, U., Meurer, M., Chuartzman, S., Zalckvar, E., Goldman, O., Ben-Dor, S., Schutze, C., Wiedemann, N., Knop, M., et al. (2016). One library to make them all: streamlining the creation of yeast libraries via a SWAp-Tag strategy. Nat. Methods 13: 371-378.
